# Germline *MC1R* status influences somatic mutation burden in melanoma

**DOI:** 10.1038/ncomms12064

**Published:** 2016-07-12

**Authors:** Carla Daniela Robles-Espinoza, Nicola D. Roberts, Shuyang Chen, Finbarr P. Leacy, Ludmil B. Alexandrov, Natapol Pornputtapong, Ruth Halaban, Michael Krauthammer, Rutao Cui, D. Timothy Bishop, David J. Adams

**Affiliations:** 1 Experimental Cancer Genetics, The Wellcome Trust Sanger Institute, Hinxton, Cambridgeshire CB10 1SA, UK; 2 Laboratorio Internacional de Investigación sobre el Genoma Humano, Universidad Nacional Autónoma de México, Campus Juriquilla, Boulevard Juriquilla 3001, Santiago de Querétaro 76230, Mexico; 3 The Cancer Genome Project, The Wellcome Trust Sanger Institute, Hinxton, Cambridgeshire CB10 1SA, UK; 4 Department of Pharmacology and Experimental Therapeutics, Boston University School of Medicine. Boston, Massachusetts 02118, USA; 5 MRC Biostatistics Unit, Cambridge Institute of Public Health, Cambridge Biomedical Campus, Cambridge CB2 0SR, UK; 6 Division of Population Health Sciences, Royal College of Surgeons in Ireland, Lower Mercer Street, Dublin 2, Ireland; 7 Department of Pathology, Yale University School of Medicine, New Haven, Connecticut 06519, USA; 8 Department of Dermatology, Yale University School of Medicine, New Haven, Connecticut 06519, USA; 9 Program in Computational Biology and Bioinformatics, Yale University School of Medicine, New Haven, Connecticut 06519, USA; 10 Section of Epidemiology and Biostatistics, Leeds Institute of Cancer and Pathology, University of Leeds, Leeds, LS9 7TF, UK

## Abstract

The major genetic determinants of cutaneous melanoma risk in the general population are disruptive variants (*R* alleles) in the melanocortin 1 receptor (*MC1R*) gene. These alleles are also linked to red hair, freckling, and sun sensitivity, all of which are known melanoma phenotypic risk factors. Here we report that in melanomas and for somatic C>T mutations, a signature linked to sun exposure, the expected single-nucleotide variant count associated with the presence of an *R* allele is estimated to be 42% (95% CI, 15–76%) higher than that among persons without an *R* allele. This figure is comparable to the expected mutational burden associated with an additional 21 years of age. We also find significant and similar enrichment of non-C>T mutation classes supporting a role for additional mutagenic processes in melanoma development in individuals carrying *R* alleles.

## Abstract (web summary)

Deleterious germline variants in the *MC1R* gene are associated with red hair and freckles, and with an increased risk of developing melanoma. Here, the authors investigate melanoma samples from patients with and without these variants and find that their presence is predictive of a higher overall mutation prevalence.

## Graphical abstract

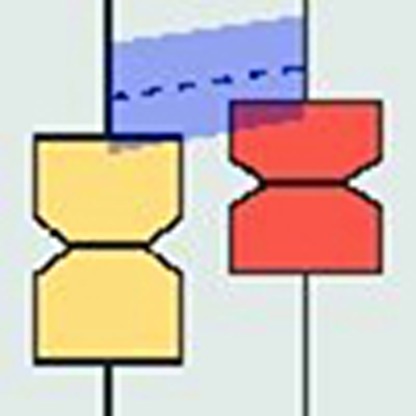

Melanocortin 1 receptor (MC1R) is a G protein-coupled receptor expressed on the surface of melanocytes that signals to downstream effectors, such as the microphthalmia-associated transcription factor, to regulate skin pigmentation and to control cell proliferation and apoptosis^1^. Melanin is generated by melanocytes in two major forms, pheomelanin and eumelanin. Unlike eumelanin, which is dark brown or black in colour, pheomelanin is red/orange, and is associated with type I/II skin, freckles, red hair and an inability to tan^2^. People with this phenotype are generally highly photosensitive, and prone to sunburn when exposed to ultraviolet (UV) light^3^.

Population sequencing studies have revealed a number of null or hypomorphic *MC1R* alleles, which are collectively referred to as *R* alleles and are strongly associated with the red hair and light skin phenotype^4^,^5^. Other missense variants are referred to as *r* alleles and are less strongly associated with this hair colour and complexion^4^,^6^. While *R/R* individuals are generally red heads, and persons with 0 or 1 *R* alleles are rarely red heads, pigmentation traits such as degree of tanning after repeated UV exposure^3^ and skin reflectance^7^ depend additively on the number of *R* alleles. In addition to regulating skin pigmentation, MC1R signalling has been reported to increase phosphorylation of DNA repair proteins, initiating the DNA damage repair process, and also to activate survival pathways^8^,^9^,^10^,^11^. As such, polymorphisms in *MC1R* have been linked to increased melanocyte apoptosis and inefficient DNA repair^12^. Collectively these factors link *MC1R* variants to increased melanoma risk^6^,^13^,^14^. In animal model systems, null alleles of *Mc1r* (*Mc1r*^*e/e*^) have been shown to co-operate with *Braf*^*V600E*^ to promote melanoma development via mechanisms including enhanced lipid peroxidation, a phenotype rescued on an albino mouse background owing to a lack of pheomelanin production^15^. This has led to the suggestion that loss of MC1R function, even in the absence of UV light, may be oncogenic. Despite these insights, it is still unclear whether *MC1R* germline variant alleles influence the genome-wide somatic mutation burden in melanoma.

In this study, we set out to establish the contribution of germline *MC1R* alleles to the somatic mutation landscape of sporadic melanoma. Mutations found in melanomas are predominantly C>T transitions due to the production of cyclobutane pyrimidine dimers (CPDs) in response to solar UV damage, but other mutational classes such as C>A transversions have also been observed^16^,^17^,^18^,^19^,^20^. Indeed, hotspot mutations in key driver genes, such as *BRAF* and *KIT*, are almost exclusively acquired as non-C>T mutations^17^. Our results indicate that individuals with one or two germline *MC1R R* alleles have a significantly higher somatic mutational load than individuals with no *R* alleles. This finding has implications for our understanding of melanomagenesis, as well as the identification of individuals at higher risk of developing melanoma.

## Results

### Samples

We analysed somatic single-nucleotide variants (SNVs) from two independent melanoma cohorts: melanoma samples from The Cancer Genome Atlas (TCGA) skin cutaneous melanoma (SKCM) collection^21^, and a data set from the Yale Melanoma Genome Project^22^. We studied only cases with stated ethnicity ‘white' and with a histopathological diagnosis of cutaneous melanoma (excluding acral and mucosal melanoma which are not thought to be UV related). The TCGA data set included 273 tumour/germline exome pairs (out of the total collection of 343 samples), which comprised 43 from primary tumours and 230 from metastases (all from different patients) (Methods section, Supplementary Data 1 and 2). The Yale data set was composed of 132 tumour/germline exome pairs, all of which were from whites with a confirmed diagnosis of cutaneous melanoma (28 primaries, 104 metastases) (Supplementary Data 3).

### Variant calling and definition of SNV sets

To define *MC1R* genotypes for each patient we analysed germline binary alignment/map (BAM) files to identify and classify all non-synonymous and nonsense variants as either *R, r* or wild-type alleles using the method shown in Supplementary Fig. 1, and which is described in the Methods section. Since *MC1R* disruptive variants segregate on separate haplotypes, we assumed persons with two *R* alleles to be homozygotes or compound heterozygotes. Sample numbers for each *MC1R* genotype are shown in Supplementary Fig. 2, with additional details found in Supplementary Data 2 and 3, and Supplementary Table 1. In the TCGA cohort, 23 (8%) individuals had two *R* alleles, 112 (41%) individuals had one *R* allele, and 138 (51%) individuals had genotypes that were *0/0*, *r/0* or *r/r* (zero *R* alleles). In the Yale data set, five (4%) individuals had two *R* alleles, 54 (41%) individuals had 1 *R* allele, and 73 (55%) individuals had 0 *R* alleles.

We downloaded somatic variant calls from the TCGA that had been previously generated by three pipelines; the Broad Institute MuTect pipeline (version 119)^23^, the Baylor College of Medicine CARNAC pipeline (version 1.0), and the bambam pipeline from University of California at Santa Cruz (UCSC; version 1.4). To generate a stringent call set for downstream analysis, we selected these previously generated SNVs that were agreed on by two or three of these pipelines (Methods section, Supplementary Fig. 3). Somatic variants from the Yale data set were generated as described previously^22^. For our analysis we counted double and triple-nucleotide variants separately as SNVs. These calls accounted for an average of 4.9% of all somatic variants (range 0–18%).

### Modelling somatic SNV burden against *R* allele presence

For each of the six basic SNV classes (C>A, C>G, C>T, T>A, T>C and T>G), we modelled the relationship between expected somatic SNV burden and the presence of *MC1R R* alleles using negative binomial regression (Methods section). For the TCGA cohort, additional predictor variables were included to control for age, sex, sample type (primary or metastasis), tissue collection centre, body area, Breslow thickness, Clark's level and ulceration status (Methods section, Supplementary Data 2). Missing values in these predictor variables (∼12% of the data) were multiply imputed (Methods section). The first three predictor variables were available for the Yale data set (Supplementary Data 3), with no missing information.

**Figure 1:**
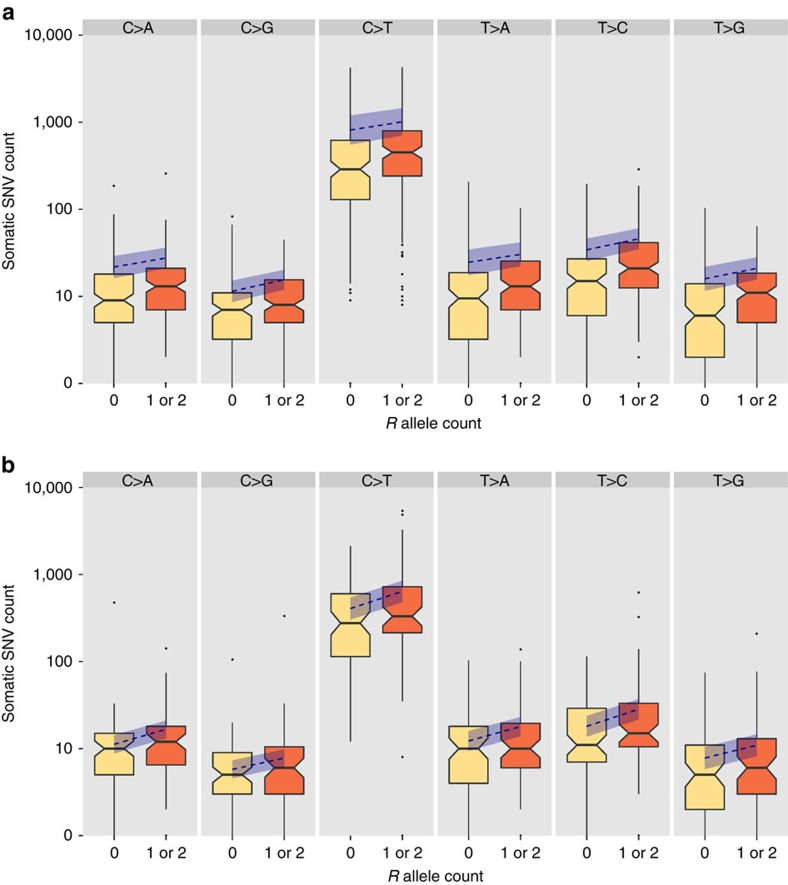
The distribution of SNV counts detected through exome sequencing of melanoma samples. SNV counts are grouped by the presence of R alleles of the *MC1R* locus, and are shown as a boxplot with median, quartiles, whiskers and outliers. For each SNV class, the blue dashed line (and ribbon) charts the predicted mean mutation burden (and 95% confidence interval) of a patient with the most common constellation of values for clinical variables as the *R* allele count increases from zero to one or two, with all other clinical variables held fixed. (**a**) TCGA melanoma cohort, in which the most common constellation represents a 57-year-old male from the University of Sydney collection centre, with a metastasis to a regional lymph node of the extremities and a primary Breslow depth of 2.5 mm, Clark level IV, and no ulceration. Please note that one sample, TCGA-FW-A3R5, with *R* genotype 0/0 is an outlier with more than 10,000 C>T somatic mutations and is not depicted in the C>T panel of this image. (**b**) Yale Melanoma Project cohort, in which the most common constellation represents a 65 year-old male sampled via a metastasis.

Figure 1a shows the distribution of the somatic SNV counts per tumour in the TCGA melanoma cohort broken down by *R* allele presence, and Supplementary Table 2 shows the results of the negative binomial regression adjusted for the above-mentioned eight clinical variables. Figure 1b and Supplementary Table 3 show the equivalent results for the Yale data set adjusted for the three available clinical variables. For all six mutation types in both data sets, there was evidence of an increased burden of somatic SNVs in persons carrying one or two *R* alleles. After Benjamini–Hochberg correction for testing for six mutation classes, statistically significant (*P*<0.05) differences in expected SNV counts were observed in four of the six mutation classes in the TCGA data set (Table 1, Supplementary Table 2), and in five out of six in the Yale data set (Table 1, Supplementary Table 3). Statistically significant differences in expected SNV count were observed for all six mutation classes in the combined data set (all adjusted *P*<0.01, Table 1, Supplementary Table 4), with this analysis being performed adjusting for the clinical variables common to both collections. For fixed values of the other predictors and for the C>T mutation class, carrying one or more *R* alleles was associated with a 24% (95% confidence interval (CI): −5 to 63%) increase in the expected somatic SNV count in the TCGA data set, a 58% (95% CI: 11–124%) increase in the Yale data set, and a 42% (95% CI: 15–76%) increase in the combined data set (Table 1, Supplementary Table 4). Aside from *MC1R* genotype, for the C>T mutation class in the combined data set, the other significant predictors of mutation burden were age (each extra year was associated with a 1.7% (95% CI: 0.9–2.4%) increase in the expected somatic SNV count) and tissue collection centre (Supplementary Table 4). Compared with the ‘baseline' collection centre at the University of Sydney, melanomas from several other centres had significantly lower C>T mutational burdens, namely MD Anderson Medical Center, Greater Poland Cancer Centre, the University of Pittsburgh, Essen and Yale, whereas melanomas from The International Genomics Consortium had significantly higher numbers of this class of mutation (Supplementary Table 4). For the TCGA data set, a location on the head and neck rather than the extremities and tissue collection centre were additional significant predictors of C>T somatic mutation burden (Supplementary Table 2). Results were similar for other mutation classes.

### Alternative models for *R* allele presence and SNV count

We also considered alternative frameworks to model the relationship between *R* allele presence and somatic SNV count. Further analyses did not reveal any consistent patterns in expected mutation counts between individuals with one as compared with two *R* alleles (Methods section, Supplementary Fig. 4). A regression analysis in the combined data set considering the total number of SNV mutations instead of SNV mutation classes also found *MC1R* genotype to be a significant predictor of mutation count, along with age of diagnosis and tissue collection centre (Methods section, Supplementary Fig. 5), a result consistent with our other analyses.

### Comparison of mutational signature differences

To further investigate the mutational differences between *R* allele carriers and non-carriers, we examined somatic mutational signatures in a trinucleotide context^24^ (Methods section). Eight mutational signatures explained 97.5% of mutations in the combined TCGA and Yale melanoma data set (Supplementary Figs 6 and 7). The sample group with one or two *MC1R R* alleles had a significantly lower prevalence of the signature linked to age-associated 5-methylcytosine deamination, a result presumably explained in part by their younger age at diagnosis (57.6 versus 62.2 years on average; Supplementary Table 5). No other significant differences in the mutational signatures were identified^24^,^25^.

### Investigation of the role of *MC1R* in melanocyte biology

**Figure 2:**
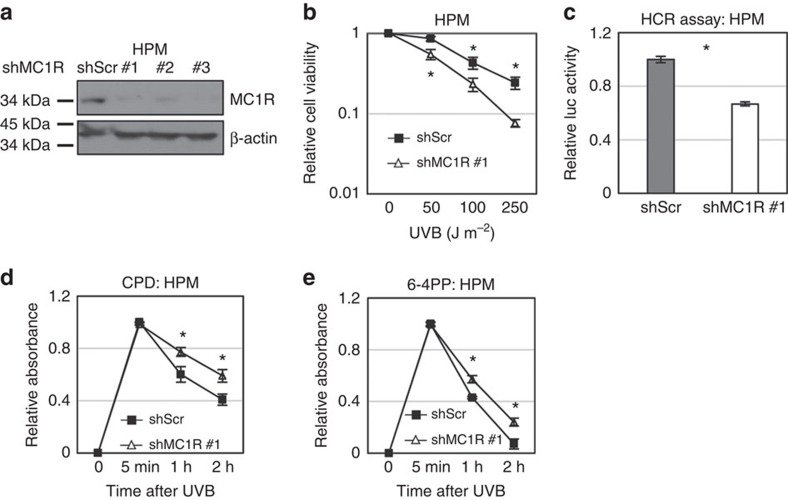
*MC1R* expression regulates the formation of mutagenic photoproducts. (**a**) Shown are HPMs stably expressing control shRNA (shScr) or multiple independent shMC1Rs targeting *MC1R* (#1, #2, #3). Cells were pre-incubated with 1 μM α-MSH for 30 min, then exposed to 100 J m^−2^ UVB light, and harvested 3 h later for Western blot analysis. (**b**) HPM cells stably expressing shMC1R#1 were irradiated with different doses of UVB light as indicated. Cells were collected at 24 h after UVB light irradiation and viability assessed using the 3-(4,5-dimethylthiazol-2-yl)-2,5-diphenyltetrazolium bromide (MTT) assay. (**c**) HPMs stably expressing an shRNA against *MC1R* (shMC1R#1) or a scrambled control (ShScr) were transfected with 2 μg of UV damaged pGL3 luciferase expression vector and 0.5 μg pRL Renilla Luciferase control reporter vector before the host cell reactivation assay^28^,^32^ (Methods section; **d,e**). HPMs stably expressing an shRNA against *MC1R* (shMC1R#1) or a scrambled control (ShScr) were irradiated with 100 J m^−2^ UVB light and then collected at the different time points indicated. Genomic DNA was extracted and photoproducts were detected by ELISA. Cyclobutane pyrimidine dimer (CPD) (**d**) or 6–4 pyrimidine photoproduct (6–4PP) (**e**) antibodies were used (Methods section). These data were compiled from three separate experiments performed in triplicate. Significance (*P<0.05) was calculated by using the unpaired two-tailed Student's *t*-test comparing the means (shScr vs shMC1R#1) of the three experiments. Error bars represent s.d.

We next considered whether the increased number of mutations observed in tumours from *MC1R* variant carriers could be due to differential DNA repair ability in primary human melanocytes (HPMs). Therefore, we elected to explore the role of *MC1R* in HPM cultures in response to UV light (Methods section, Fig. 2). *MC1R* wild-type melanocytes were transfected with short hairpin RNA (shRNA) constructs resulting in the suppression of *MC1R* expression, or with a scrambled control shRNA, thus creating an isogenic pair of cell lines for functional studies. Importantly, knockdown of *MC1R* reduced but did not abolish *MC1R* expression, analogous to MC1R expression in *R* allele heterozygotes^26^,^27^ (Fig. 2a, Supplementary Fig. 8). Irradiation of cell lines with escalating doses of UV light (302 nm) resulted in significantly reduced survival of *MC1R* knockdown cells compared with cells transfected with a scrambled shRNA control (Fig. 2b), and reduced activity of the transcription-coupled/nucleotide excision repair pathway, as evidenced by reduced luciferase activity in the host cell reactivation assay^28^ (Fig. 2c). Reduced *MC1R* expression was also associated with increased levels of CPDs (Fig. 2d) and 6–4 pyrimidine photoproducts (6–4PP, Fig. 2e), a result in keeping with genetic models using melanocytes from *R* allele carriers^8^,^10^.

## Discussion

Most mutations found in melanoma genomes are likely to be passengers, and are thus reflective of the UV exposure and other mutagenic processes operative over a patient's lifetime. Our regression analyses suggest that the estimated increase in expected SNV count associated with the presence of an *MC1R R* allele for the C>T mutation class is comparable to the estimated increase associated with an additional 21 years of age in the combined data set, with similar estimates across the other mutation classes (range 18–29 years; Table 1). *R* allele presence is also a significant predictor of total SNV count (Supplementary Fig. 5). Interestingly, our analysis did not reveal any consistent patterns in mutation counts between individuals with one as compared with two *R* alleles (Methods section, Supplementary Fig. 4). This suggests that the majority of persons with one *R* allele, who do not have a red hair/sun sensitivity phenotype, may still be highly susceptible to the mutagenic effects of UV light. Of note, it has been suggested that red haired, sun-sensitive individuals are more likely to practice sun avoidance^29^,^30^, a factor that confounds ready interpretation of the association between mutation count and number of *R* alleles.

Our study finds that melanomas from individuals carrying *MC1R R* variants associated with red hair and freckling have a significantly higher somatic mutational burden than melanomas from individuals with no *MC1R R* variants. Intriguingly, while C>T mutations were the most common mutational class observed across all *MC1R* genotypes, all mutation classes were significantly and similarly increased. This might reflect a form of ‘collateral damage' resulting from a decreased ability of cells in patients with *R* alleles to protect themselves from UV damage, or indicate that other mutational processes are operative in melanocytes from these patients. When we compared the mutational signatures present in *MC1R R* allele carriers to those present in non-carriers (Supplementary Figs 6 and 7), we observed no significant differences between the two groups apart from the age-associated 5-methylcytosine deamination, a result explained in part by the marginally younger average age of *MC1R R* allele carriers. This suggests that the same mutational processes are operative in *R* allele carriers as in non-carriers, but that the relative frequency of mutational events may be what differs between these groups. However, studies with larger sample sizes should be performed to further investigate this relationship.

Notably, studies in mice have proposed a non-UV path to skin tumourigenesis via pheomelanin, which has been shown to induce oxidative stress and lipid peroxidation^15^. Previous studies have also investigated the role of *MC1R* in the DNA damage response in melanoma cell lines, or in primary mouse cells in culture^31^,^32^,^33^. Since DNA repair may be different in these systems when compared to non-transformed human melanocytes, we elected to explore the role of *MC1R* in HPM cultures in response to UV light. These experiments show that, in this system, *MC1R* knockdown significantly impairs survival and DNA repair (Fig. 2a–c), and was also associated with increased levels of CPDs (Fig. 2d) and 6–4PPs (Fig. 2e). While CPDs and 6–4PP are primarily associated with C>T and CC>TT mutations, they have also been associated with non-C>T mutations^19^, and thus may contribute to the elevated levels of these mutations observed in *R* allele carriers. Importantly, a recent study has shown that sequence context markedly affects the mutagenic effects of 6–4PP, and the resulting nucleotide change^34^. Notably, N>T mutations have been observed^19^, and this may provide a mechanistic explanation for the enrichment of these mutations observed in our study. Thus, the enrichment of the non-C>T signatures we observe are likely to be the result of multiple mutagenic processes such as lipid peroxidation and ROS activity^15^, together with the effects of ultraviolet photoproducts such as 6–4PP.

In summary, we find a role for germline *MC1R* variants in influencing the somatic mutational landscape of melanoma. Since red heads comprise around 1–2% of the world's population, yet 16% of melanoma patients^35^, and 26–40% of melanoma patients are *R* allele carriers^36^, this work has significant implications for understanding the genesis of melanoma in these high-risk groups.

## Methods

### Clinical variables in TCGA cohort

For the TCGA melanoma cohort^21^, the available clinical variables included age at diagnosis, gender, tissue collection centre, body area, tissue type, Breslow depth, Clark level and ulceration status (Supplementary Data 2). The median age was 57 years (interquartile range 46–70, missing information for one sample), and the cohort included 99 females and 174 males. 112 samples were from the extremities, 106 from the trunk, 21 from the head and neck, 4 recorded as ‘other' and 30 were unknown. 154 samples were from a regional lymph node, 45 from regional tissue, 43 from a primary tumour and 31 from a distant metastasis. 35 samples had Clark level V, 92 level IV, 51 level III, 13 level II, five level I and 77 with unknown Clark level. 85 samples were ulcerated, 98 had no ulceration and 90 were unknown. The median Breslow depth was 2.5 mm (interquartile range 1.2–5.0, unknown for 66 samples). Tissue collection centres were: The University of Sydney (EE) which contributed 86 samples, MD Anderson (D3) 55, Essen (FS) 36, the University of Pittsburgh (ER) 30, Asterand (EB) 27, the Greater Poland Cancer Center (D9) 11, Roswell (GN) 11, the University of North Carolina (FR) 6, The International Genomics Consortium (FW) 5, ABS - IUPUI (GF) 3, Cureline (BF) 2 and The University of Miami (IH) 1.

### Germline *MC1R* variant identification

Common *MC1R* variants have previously established classifications^26^,^37^,^38^. To identify the precise variants carried by each individual, for each normal/germline BAM, we called variants in the *MC1R* region with samtools mpileup –Dsu -C50 -m2 -F0.0005 -d1000 and bcftools view -p 0.99 -vcgN, applying the standard set of vcf-annotate filters, and applying the Ensembl Variant Effect Predictor^39^ to the canonical *MC1R* transcript (ENST00000555147) including SIFT^40^ and PolyPhen^41^ scores. For each normal BAM, we extracted *MC1R* polymorphisms that passed all filters, including the nine canonical *MC1R* missense polymorphisms, nonsense or frameshift mutations and variants predicted to be deleterious or damaging by SIFT or PolyPhen (details below).

To classify rare alleles of *MC1R* as *R* or *r*, we used the SIFT 4.0.5 and PolyPhen 2.2.2 algorithms as indicated in Supplementary Fig. 1. *R* genotypes for each sample are provided in Supplementary Data 2 (TCGA) and Supplementary Data 3 (Yale). Of note, a recent report^42^ quantified membrane expression levels and cAMP induction by the rare *MC1R* alleles p.Gly89Arg, p.Thr95Met, p.Asp121Glu and p.Arg213Trp, found in patients from the TCGA cohort, as well as p.Ser83Leu, in the same position as a variant found in a patient from the Yale cohort (p.Ser83Pro). This study suggested that, whereas variants p.Gly89Arg, p.Asp121Glu and p.Ser83Leu were classified correctly by SIFT and PolyPhen 2 (all as *R* alleles), p.T95M might be more accurately classified as *r* and p.Arg213Trp as *R*. We have replicated the analysis described in this paper (using the methods described below) classifying p.Thr95Met as *r* and p.Arg213Trp as *R.* The results revealed that *R* allele count was still a significant predictor of the overall SNV mutation count. Coefficient estimates, 95% CIs, and *P* values for the *R* allele count predictor for each mutation class for this analysis are provided in Supplementary Table 6.

### Identification of somatic variants calls

We downloaded VCF files containing somatic variant calls from CGHub^43^. These calls were produced by the three TCGA analysis centres: the Broad Institute, Baylor College of Medicine, and UCSC. Variants marked as ‘PASS' and called by at least two centres were included in subsequent analyses. We compared this set of calls with those released by the main analysis of the TCGA SKCM Working Group^21^ (Supplementary Fig. 3) revealing high concordance. TCGA SKCM somatic calls were downloaded on August 2015 from http://tcga-data.nci.nih.gov/docs/publications/skcm_2015/.

### Modelling the effect of *R* alleles on somatic mutation count

For each of the six basic SNV classes (C>A, C>G, C>T, T>A, T>C and T>G), we modelled the relationship between expected somatic SNV burden and the presence of *MC1R R* alleles using negative binomial regression with a log link (that is, modelling log(expected SNV count)). We controlled for all available clinical variables. Missing clinical variable values in the TCGA data set were imputed 10 times using the MICE package in R^44^,^45^, with the variables age, Breslow thickness, gender, Clark level, ulceration, tissue source, tissue type, body area, *R* allele presence and the counts for each of the six basic mutational classes being included in the univariate imputation models^44^. Values for any level of a categorical variable with <5 samples were also set to missing and imputed. For the Yale Melanoma data set, there were fewer clinical variables available but there was no missing information.

For our primary analysis, the estimates of the effect of carrying at least one *R* allele were compared with persons carrying zero *R* alleles.

### Alternative models for *R* allele presence and SNV count

As described above, we compared the somatic mutation burden of melanomas from individuals with one or two *R* alleles to individuals with zero *R* alleles. We also considered an alternative model in which the somatic mutation count in the combined data set is regressed on *R* allele count (three levels) instead of presence/absence of an *R* allele (two levels; Supplementary Fig. 4). In this framework, the average increase in the expected C>T somatic SNV count associated with the presence of each *R* allele is comparable to the increased mutational burden associated with an additional 16.15 years of age. However, the numbers of *R*/*R* individuals in these cohorts is not sufficient to assess the contribution of *R* alleles individually.

We also considered a model in which total somatic SNV count in the combined data set is regressed on the presence or the absence of *R* alleles, instead of considering each mutation class separately (Supplementary Figure 5). This analysis is dominated by C>T mutations as they are much more abundant than the other mutational classes. The significant predictors of total somatic SNV count in this analysis were age at diagnosis, *R* allele presence and tissue collection centre.

### Extraction of mutational signatures

We extracted mutational signatures in the trinucleotide context^24^ *de novo* on the combined TCGA and Yale data sets using a Hierarchical Dirichlet Process^46^ (HDP) with one child-DP node per sample, one parent-DP node per *R* allele group (zero or at least one *R* allele), and one grandparent-DP node with a uniform Dirichlet prior distribution. We initialized the HDP with four clusters, then ran four independent Markov chain Monte Carlo (MCMC) chains with 10,000 burn-in iterations and 500 posterior samples collected off each chain with 50 iterations between each (2,000 posterior samples in total). We performed all analyses with the ‘hdp' R package version 0.1.0, available at github.com/nicolaroberts/hdp.

### Melanocyte cultures and ultraviolet exposure

HPMs were isolated from foreskins as described previously^47^ and cultured in Medium 254 from Life Technologies (Thermo Fisher Scientific, Waltham, MA) supplemented with 10% fetal bovine serum. Cells were washed with PBS twice and exposed to UVB light in a Stratalinker ultraviolet chamber (Stratagene, Cedar Creek, TX) fitted with ultraviolet B bulbs (UVP Inc. CA). Ultraviolet emittance was measured using an ultraviolet photometer (UV Products, Upland, CA, USA). An ultraviolet B dose of 100 J m^−2^ is equivalent to one standard erythema dose of UVB light.

### Plasmids and shRNA constructs

shRNA constructs targeting human MC1R (Cat. No. RHS4533-EG4157) were purchased from Open Biosystems (Thermo Fisher Scientific). The target sequence for shMC1R#1 is 5′- AAATGTCTCTTTAGGAGCCTG -3′.

### Antibodies and western blot

Anti-MC1R (N-19; at a dilution of 1:500) antibody and Peroxidase-conjugated anti-goat secondary antibody (at dilution 1:2,000) were purchased from Santa Cruz Biotechnology, Inc. (Dallas, TX). Peroxidase-conjugated β-actin (ACTB, at dilution 1:10,000) antibody was purchased from Sigma-Aldrich (St. Louis, MO). Western blot analysis was performed as described previously^47^.

### DNA damage repair assays

CPD and 6–4PP ELISA were performed by using anti-CPD and anti-6–4PP antibodies, the purified anti-mouse IgG mAb was diluted to 2 μg ml^−1^ in PBS and added into an enhanced protein-binding ELISA plate (for example, Falcon Labware plate). Antibodies specific for CPD (MC-062) and 6–4PP (KTM-50) were diluted 1:1,000 in blocking buffer and added to each well. Optical density at 405 nm was measured. For the host cell reactivation assay^28^, pGL3 (Promega, Madison, WI) was irradiated at a dose of 700 J m^−2^ of UVC light to induce DNA damage in the form of CPD and 6–4PP, which block transcription until repaired. Damaged pGL3 was transfected into cells using lipofectamine 2000 (Thermo Fisher Scientific). A *Renilla* vector was used as a transfection control.

### Data availability

Data referenced in this study are available from TCGA melanoma data set: https://cghub.ucsc.edu/, filter by disease=SKCM, analyte type=DNA, study=TCGA, sample type=Blood derived normal (for germline DNA) or Metastatic (06), Primary solid tumour (01) for tumour samples, platform=Illumina, state=live, library type=WGS, WXS. Centre=BI was selected for germline DNA analysis. The Yale data set is available from dbGAP under accession code phs000933. The authors declare that all other data supporting the findings of this study are available within the article and its Supplementary Information files or available from the authors on request. Signature analyses were performed with the ‘hdp' R package version 0.1.0, available at github.com/nicolaroberts/hdp. All scripts used to perform the analysis in this study is released as Supplementary Software 1.

## Additional information

**How to cite this article:** Robles-Espinoza, C.D. *et al*. Germline MC1R status influences somatic mutation burden in melanoma. *Nat. Commun.* 7:12064 doi: 10.1038/ncomms12064 (2016).

## Supplementary Material

Supplementary Information Supplementary Figures 1-8, Supplementary Tables 1-6 and Supplementary References.

Supplementary Data 1 All melanoma samples (SKCM) in the TCGA collection. Samples in red have been excluded from this analysis, and the reason is indicated in column K ("reason for leaving out").

Supplementary Data 2 All TCGA melanoma samples included in this study with their clinical covariates, MC1R genotype and mutation counts in trinucleotide context. For coding of Column G (tissue_source_site), see Methods.

Supplementary Data 3 All melanoma samples part of the Yale Melanoma Project with their covariates, MC1R genotype and mutation counts in trinucleotide context. In column D, F=Female, M=Male.

Supplementary Software 1 Hierarchical Dirichlet Process package for R

## Figures and Tables

**Table 1:**
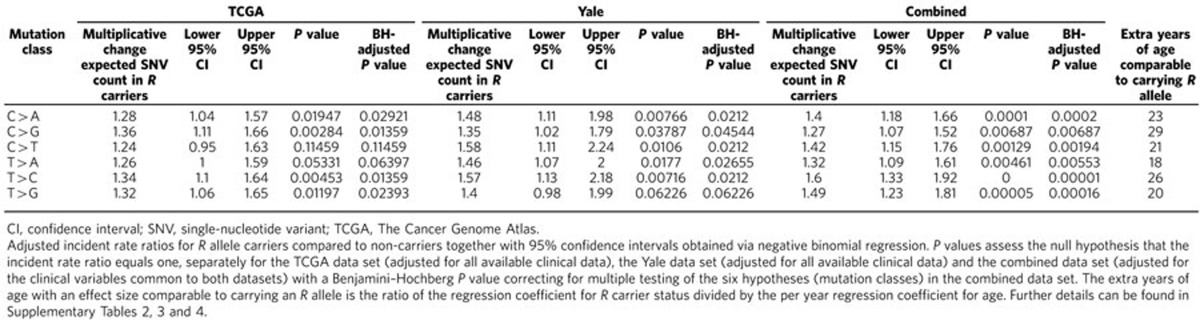
Estimated multiplicative change in expected SNV count by *R* allele status adjusted for clinical and demographic variables.
