# Supplementary material for: Germline MC1R status influences somatic mutation burden in melanoma
Source: Nat Commun. 2016 Jul 12;7:12064. doi: 10.1038/ncomms12064 (PMC4945874; doi:10.1038/ncomms12064)
Supplement: Supplementary Information — Supplementary Figures 1-8, Supplementary Tables 1-6 and Supplementary References. [file ncomms12064-s1.pdf]

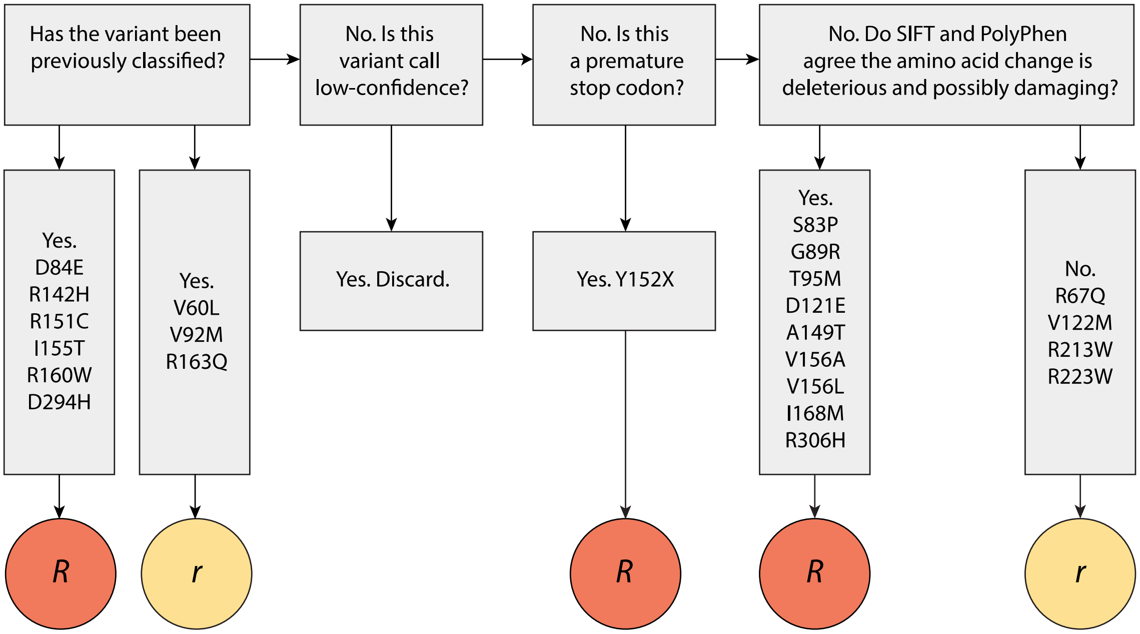

Supplementary Figure 1: Classification scheme for non-synonymous and nonsense germline *MC1R* variants. The common variants with previously established classifications[1–3] are shown. The effect of novel missense mutations were estimated by SIFT[4] and PolyPhen-2[5] through the Variant Effect Predictor[6]. See **Methods** for additional information.

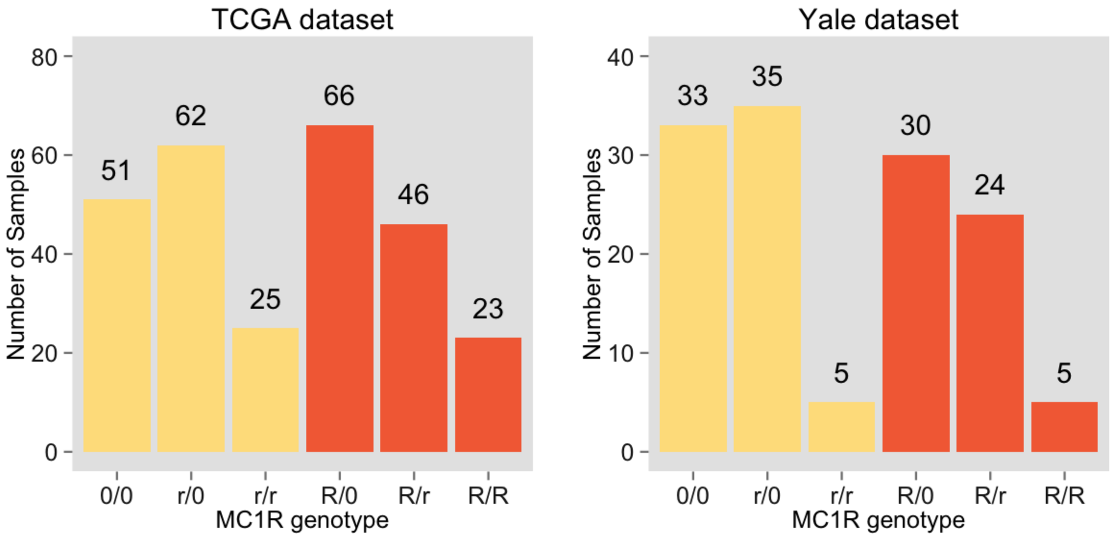

Supplementary Figure 2: The number of individuals analysed in the present study by *MC1R* genotype. The red-hair/fair-skin phenotype is strongly associated with the *R* alleles, weakly associated with the *r* alleles, and not associated with *0* (wild-type) alleles. Each *MC1R* genotype category for the TCGA and Yale Melanoma Project dataset is coloured according to the presence (or absence) of *R* alleles.

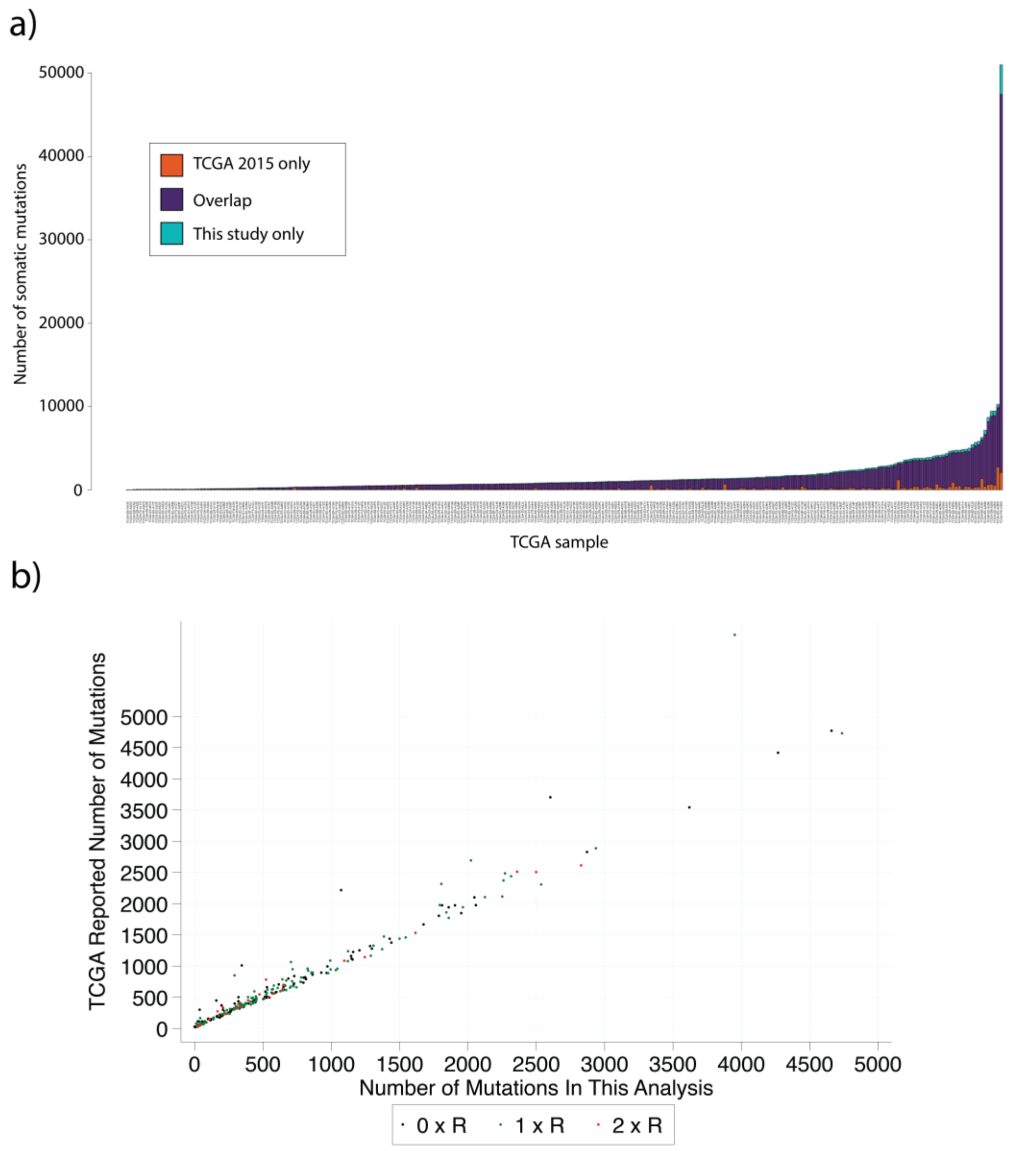

Supplementary Figure 3. Comparison of the somatic calls per sample used in this study with that one released by the TCGA SKCM Working Group[7]. a) The height of each bar represents the sum of calls in both the TCGA study and this study, and the proportion of overlapping and non-overlapping calls is indicated per sample (orange, TCGA only; violet, both TCGA and this study; blue, this study only). SNV calls are compared by position. X axis: TCGA sample ID, Y axis: number of somatic calls. b) Within the scatter plot, each point refers to TCGA sample with the number of mutations reported by the TCGA on the y-axis while on the x-axis are the number of mutations from the pipeline adopted in this analysis (see **Methods**). The pairwise correlation between the two measures is 0.97 showing the overall similarity of the two pipelines. Each sample is coloured according to the number of *MC1R* "*R*" alleles calculated for that sample. Please note that one sample, TCGA-FW-A3R5, is an outlier with more than 20,000 somatic mutations and is not depicted in this panel.

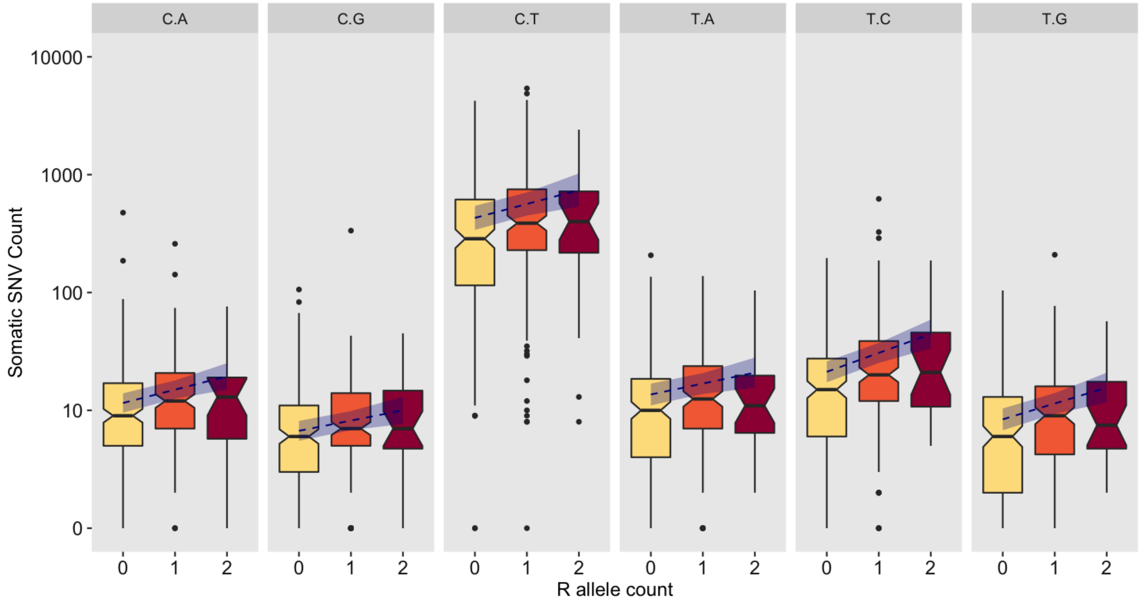

Supplementary Figure 4. The distribution of SNV counts detected through exome sequencing of melanoma samples from the combined dataset (TCGA & Yale)[7,8], grouped by *R* allele count of the *MC1R* locus. For each SNV class, the blue dashed line (and ribbon) charts the predicted mean mutation burden (and 95% confidence interval) of a patient with the most common constellation of values for clinical variables as the *R* allele count increases from zero to one to two, with all other clinical variables held fixed in the most common constellation.

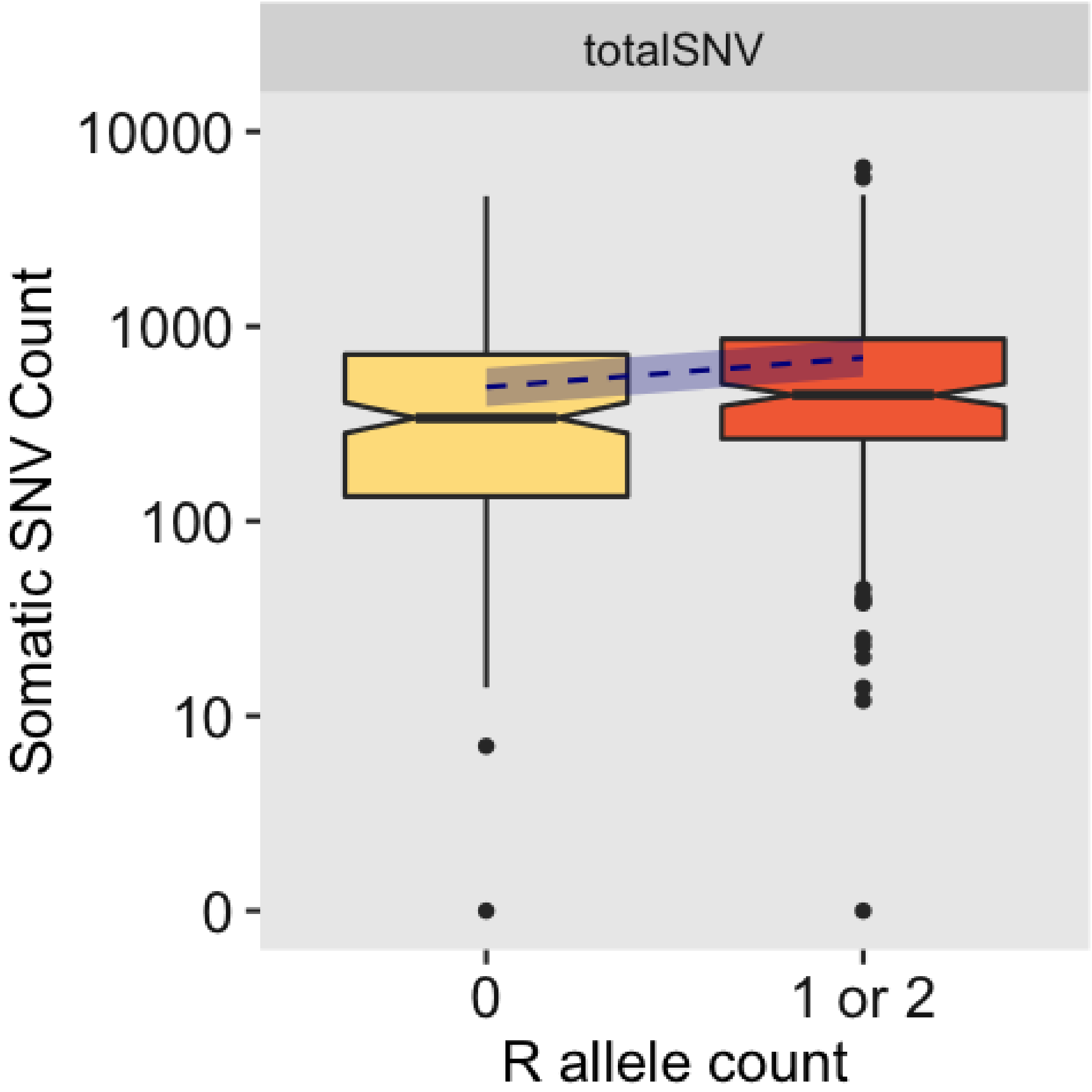

Supplementary Figure 5. The distribution of total SNV counts detected through exome sequencing of melanoma samples from the combined dataset (TCGA + Yale)[7,8], grouped by *R* allele presence of the *MC1R* locus. The blue dashed line (and ribbon) charts the predicted mean mutation burden (and 95% confidence interval) of a patient with the most common constellation of values for clinical variables as the *R* allele count increases from zero to one or two, with all other clinical variables held fixed in the most common constellation.

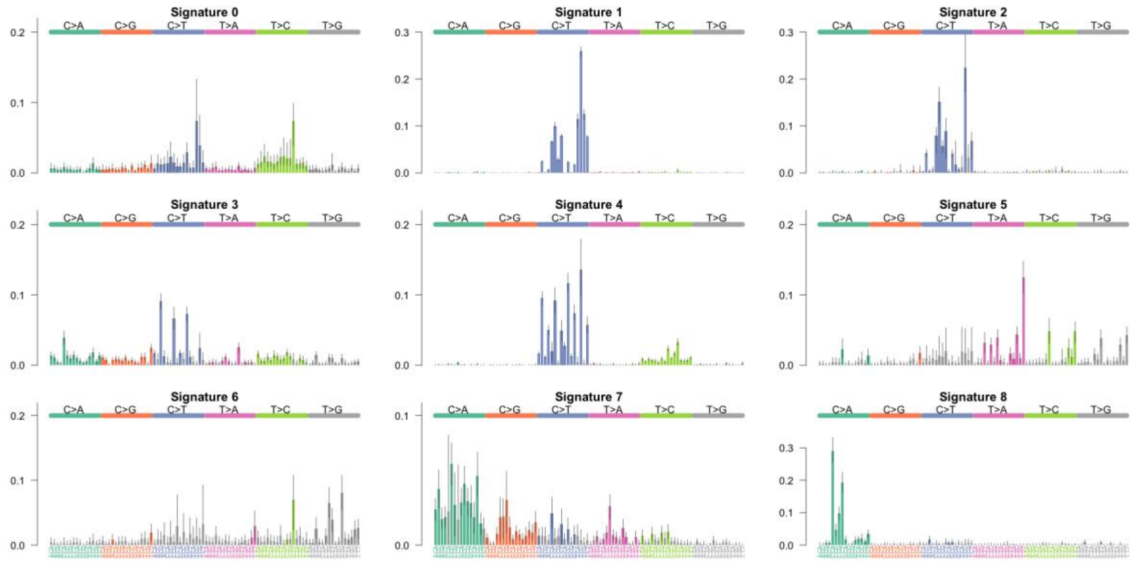

Supplementary Figure 6. Mutational signatures over the 96 base substitution classes in the trinucleotide context, each given in reference to the pyrimidine base. Eight mutational signatures explain 97.5% of mutations in the TCGA and Yale combined melanoma dataset. The remaining 2.5% of mutations left unexplained are assigned to Signature 0 to account for noise and uncertainty. Signatures 1 and 2 correspond to the UV radiation signature previously identified by COSMIC as signature 7[9] (cosine similarity 0.99 and 0.90 respectively). Signature 3 corresponds to COSMIC signature 1 (cosine similarity 0.88) associated with spontaneous deamination of 5-methylcytosine. Signature 4 corresponds to COSMIC signature 11 (cosine similarity 0.95) associated with alkylating agents (e.g. temozolomide treatment). Signatures 5 and 6 have no close match in the COSMIC database. Signature 7 corresponds to COSMIC signature 4 (cosine similarity 0.91) associated with tobacco. Signature 8 matches closely with the sequencing artefact signature R2 described by Alexandrov et al. (2013)[10]. The thin black lines indicate the 95% credibility intervals for each probability bar.

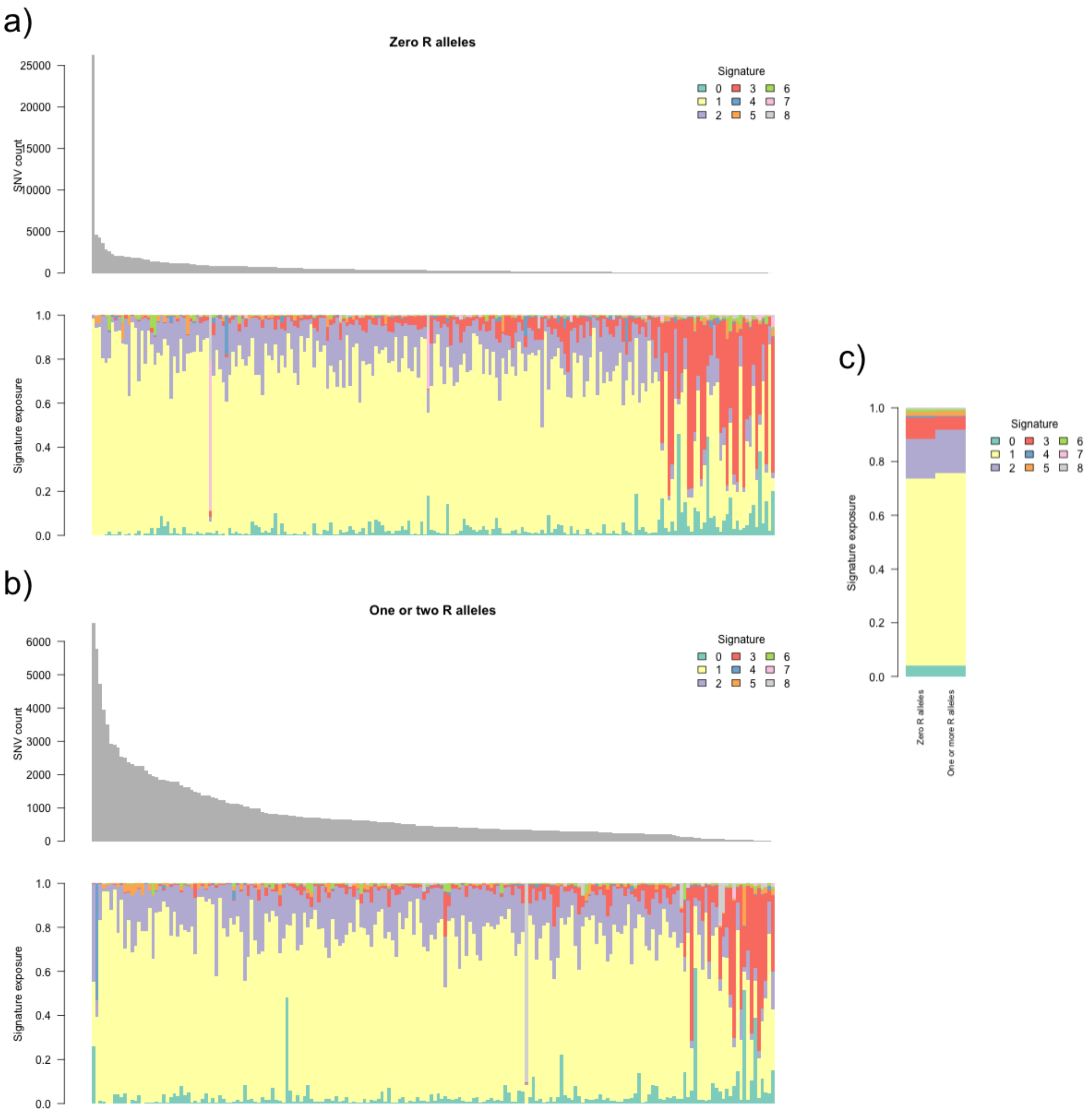

Supplementary Figure 7. Signature exposure pattern comparison between *R* allele carriers and non-carriers. a) Per-sample signature exposure patterns for samples carrying zero *R* alleles, and b) at least one *R* allele. c) Per-group signature exposure patterns, illustrating the distribution of mutational signatures in melanoma samples with zero R alleles or one or two *R* alleles.

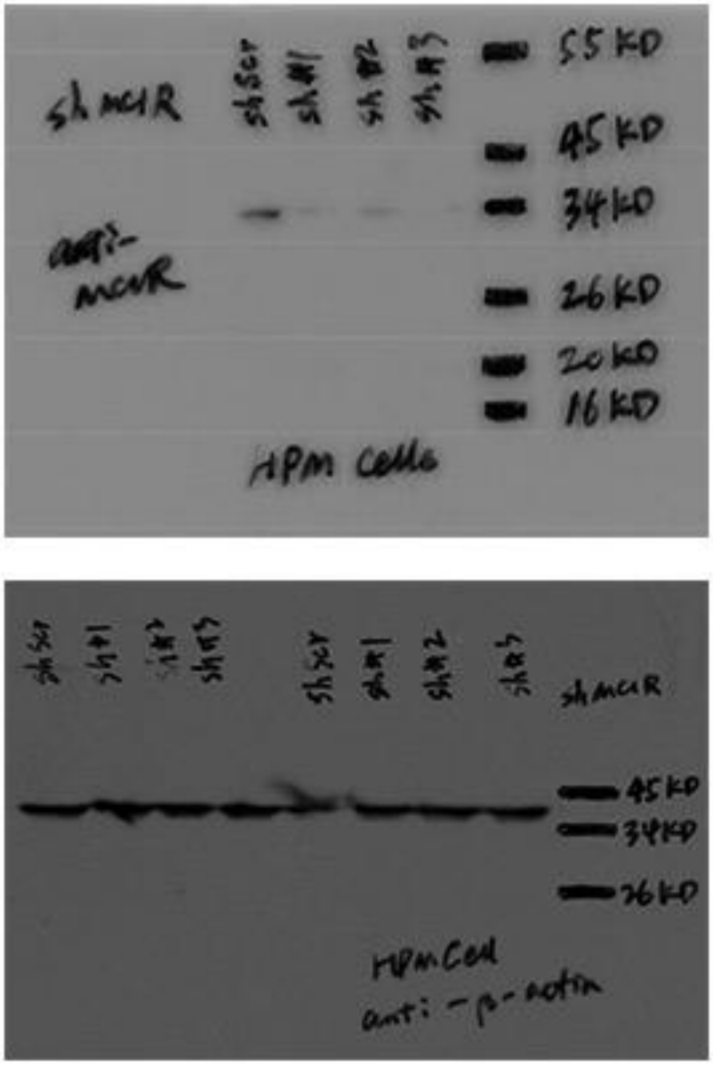

Supplementary Figure 8. Full Western Blots used to assemble **Fig. 2a**. Two repeats were stained with actin to ensure protein normalization. The molecular weight of MC1R (top panel) and actin (lower panel) is very similar so two membranes need to be stained to discern them.

Supplementary Tables

Supplementary Table 1. The number of detected alleles for each non-synonymous germline *MC1R* variant in the set of 273 TCGA samples and 132 samples part of the Yale Melanoma Project. Due to the diploid nature of the germline, up to two of these alleles can co-occur in any one sample.

Allele classification	Polymorphism	Allele count in TCGA dataset	Allele count in Yale dataset
R	Ser89Pro	0	1
	Asp84Glu	12	2
	Gly89Arg	1	0
	Thr95Met	1	0
	Asp121Glu	1	0
	Arg142His	5	3
	Ala149Thr	0	1
	Arg151Cys	58	30
	Tyr152*	1	0
	Ile155Thr	4	2
	Val156Ala	1	0
	Val156Leu	1	0
	Arg160Trp	54	15
	Ile168Met	0	1
	Asp294His	18	9
	Arg306His	1	0
r	Val60Leu	75	38
	Val92Met	59	22
	Val122Met	0	1
	Arg163Gln	23	8
	Arg213Trp	1	0
	Arg223Trp	0	1

Supplementary Table 2. Output from negative binomial regressions with the TCGA data, with separate models fitted for each of the six single nucleotide variant (SNV) classes (outcome is SNV count). In these models, the exponential of the estimated coefficient, the incident rate ratio, can be interpreted as a multiplicative factor affecting the expected SNV count outcome variable. The accompanying confidence intervals are at 95%, and stars indicate significance at thresholds: 0.10 (^), 0.05 (*) and 0.01 (**).

Variable	Categorical variable level	Outcome	Coefficient estimate	P-value	Corrected p-value (BH)	exp(Coefficient)	Confidence interval for exp(Coefficient)		
Intercept		C>A	1.45672	0.00199	0.00398	4.29184	1.74181	10.57515	
		C>G	0.83154	0.03953	0.04284	2.29686	1.04124	5.06662	
		C>T	5.24326	0.00000	0.00000	189.28581	59.25194	604.69102	
		T>A	1.56975	0.00372	0.00558	4.80543	1.69224	13.64590	
		T>C	2.15509	0.00000	0.00000	8.62870	3.75176	19.84521	
		T>G	0.93702	0.04284	0.04284	2.55235	1.03116	6.31764	
R allele presence		C>A	0.24455	0.01947	0.02921	1.27704	1.04064	1.56715	*
		C>G	0.30615	0.00284	0.01359	1.35819	1.11223	1.65853	*
		C>T	0.21819	0.11459	0.11459	1.24382	0.94811	1.63176	
		T>A	0.22955	0.05331	0.06397	1.25803	0.99671	1.58787	^
		T>C	0.29453	0.00453	0.01359	1.34250	1.09644	1.64378	*
		T>G	0.28024	0.01197	0.02393	1.32345	1.06423	1.64581	*
Age at diagnosis (years)		C>A	0.00955	0.00416	0.00757	1.00960	1.00306	1.01619	**
		C>G	0.00971	0.00433	0.00757	1.00976	1.00309	1.01648	**
		C>T	0.00709	0.11692	0.11692	1.00712	0.99821	1.01610	
		T>A	0.00935	0.02312	0.02774	1.00940	1.00130	1.01756	*
		T>C	0.00958	0.00504	0.00757	1.00962	1.00292	1.01637	**
		T>G	0.01280	0.00062	0.00372	1.01289	1.00556	1.02027	**
Breslow thickness (mm)		C>A	0.02664	0.00071	0.00213	1.02699	1.01146	1.04277	**
		C>G	0.03585	0.00011	0.00066	1.03650	1.01858	1.05474	**
		C>T	0.02462	0.06980	0.10470	1.02492	0.99793	1.05264	
		T>A	0.01734	0.17552	0.17552	1.01749	0.99190	1.04374	
		T>C	0.02062	0.03193	0.06387	1.02083	1.00182	1.04020	^
		T>G	0.01635	0.08838	0.10605	1.01648	0.99750	1.03583	
Gender (baseline level: female)	Male	C>A	0.18681	0.08826	0.17651	1.20539	0.97215	1.49461	
		C>G	0.02343	0.82239	0.82239	1.02370	0.83363	1.25711	
		C>T	0.24463	0.08635	0.17651	1.27715	0.96538	1.68960	
		T>A	0.13541	0.27980	0.34237	1.14501	0.89502	1.46481	
		T>C	0.11888	0.28531	0.34237	1.12623	0.90490	1.40171	
		T>G	0.22775	0.05051	0.17651	1.25577	0.99949	1.57775	
Clark level	Level I	C>A	0.80972	0.02907	0.17442	2.24729	1.08801	4.64177	

(baseline level: V)		C>G	0.61133	0.11405	0.23894	1.84288	0.85879	3.95464	
		C>T	0.59034	0.24072	0.24072	1.80459	0.66673	4.88440	
		T>A	0.63323	0.15929	0.23894	1.88368	0.77584	4.57346	
		T>C	0.51352	0.20183	0.24072	1.67117	0.75326	3.70764	
		T>G	0.61446	0.14663	0.23894	1.84865	0.80244	4.25889	
	Level II	C>A	0.23341	0.44705	0.54417	1.26290	0.68418	2.33112	
		C>G	0.15923	0.54417	0.54417	1.17261	0.69757	1.97116	
		C>T	0.38627	0.29531	0.54417	1.47148	0.70924	3.05290	
		T>A	0.25021	0.45963	0.54417	1.28429	0.65586	2.51488	
		T>C	0.33939	0.31115	0.54417	1.40410	0.71710	2.74925	
		T>G	0.36770	0.22880	0.54417	1.44441	0.79033	2.63983	
	Level III	C>A	0.58628	0.01070	0.06418	1.79729	1.15130	2.80575	^
		C>G	0.46024	0.02740	0.08219	1.58445	1.05372	2.38251	^
		C>T	0.53914	0.07193	0.11028	1.71453	0.95180	3.08848	
		T>A	0.54780	0.13231	0.15877	1.72944	0.83481	3.58281	
		T>C	0.28487	0.27014	0.27014	1.32959	0.79378	2.22707	
		T>G	0.46085	0.07352	0.11028	1.58543	0.95559	2.63039	
	Level IV	C>A	0.59062	0.00220	0.01322	1.80511	1.24594	2.61524	*
		C>G	0.46332	0.00969	0.02907	1.58935	1.12173	2.25190	*
		C>T	0.45968	0.06284	0.07541	1.58357	0.97509	2.57176	^
		T>A	0.53571	0.04154	0.07541	1.70866	1.02200	2.85666	^
		T>C	0.22914	0.34716	0.34716	1.25752	0.76798	2.05910	
		T>G	0.42271	0.06173	0.07541	1.52609	0.97862	2.37983	^
Ulceration (baseline level: no)	Yes	C>A	-0.21187	0.16523	0.85088	0.80907	0.59748	1.09559	
		C>G	-0.13480	0.28710	0.85088	0.87389	0.68059	1.12209	
		C>T	-0.01078	0.95129	0.95129	0.98928	0.69674	1.40465	
		T>A	-0.07016	0.70906	0.85088	0.93225	0.63684	1.36469	
		T>C	-0.10846	0.45019	0.85088	0.89721	0.67413	1.19412	
		T>G	-0.07359	0.64159	0.85088	0.92905	0.67727	1.27443	
Body area (baseline level: extremities)	Head and neck	C>A	0.48593	0.02430	0.02430	1.62568	1.06693	2.47705	*
		C>G	0.69236	0.00015	0.00044	1.99842	1.40312	2.84629	**
		C>T	0.85125	0.00186	0.00223	2.34257	1.37996	3.97669	**
		T>A	0.73610	0.00134	0.00201	2.08777	1.33714	3.25979	**
		T>C	0.89159	0.00002	0.00013	2.43900	1.63379	3.64105	**
		T>G	0.67926	0.00128	0.00201	1.97241	1.30947	2.97098	**
	Trunk	C>A	-0.27845	0.01334	0.08004	0.75696	0.60753	0.94313	^
		C>G	-0.13729	0.20194	0.20194	0.87172	0.70563	1.07690	
		C>T	-0.26137	0.10074	0.16671	0.77000	0.56308	1.05295	
		T>A	-0.24545	0.07870	0.16671	0.78235	0.59481	1.02902	

		T>C	-0.17092	0.13893	0.16671	0.84289	0.67180	1.05756	
		T>G	-0.18337	0.13713	0.16671	0.83246	0.65335	1.06067	
Tissue type (baseline level: primary tumour)	Distant metastasis	C>A	0.66806	0.01801	0.02619	1.95045	1.12336	3.38651	*
		C>G	0.70199	0.01035	0.02070	2.01777	1.18263	3.44265	*
		C>T	0.68400	0.06460	0.06460	1.98178	0.95887	4.09594	^
		T>A	0.82625	0.00899	0.02070	2.28473	1.23207	4.23677	*
		T>C	0.83591	0.00313	0.01881	2.30692	1.32989	4.00176	*
		T>G	0.67336	0.02182	0.02619	1.96082	1.10387	3.48301	*
	Regional (sub) cutaneous tissue	C>A	0.47390	0.10209	0.12251	1.60625	0.90810	2.84115	
		C>G	0.55868	0.03211	0.06676	1.74836	1.04948	2.91263	^
		C>T	0.55957	0.12546	0.12546	1.74991	0.85327	3.58877	
		T>A	0.63600	0.03743	0.06676	1.88892	1.03808	3.43711	^
		T>C	0.66664	0.01536	0.06676	1.94769	1.13810	3.33316	^
		T>G	0.58446	0.04450	0.06676	1.79403	1.01461	3.17217	^
	Regional lymph node	C>A	0.22973	0.38593	0.38593	1.25826	0.74520	2.12454	
		C>G	0.48789	0.05484	0.23015	1.62888	0.98976	2.68070	
		C>T	0.30621	0.36410	0.38593	1.35827	0.69799	2.64315	
		T>A	0.42732	0.15344	0.23015	1.53314	0.85083	2.76261	
		T>C	0.40350	0.11692	0.23015	1.49706	0.90292	2.48216	
		T>G	0.42566	0.11212	0.23015	1.53060	0.90453	2.59000	
Tissue collection centre (baseline level: University of Sydney)	D3 (MD Anderson)	C>A	-0.42346	0.00375	0.00450	0.65478	0.49255	0.87044	**
		C>G	-0.38739	0.00766	0.00766	0.67883	0.51126	0.90132	**
		C>T	-0.77339	0.00005	0.00008	0.46145	0.31861	0.66833	**
		T>A	-0.71115	0.00003	0.00007	0.49108	0.35266	0.68382	**
		T>C	-0.72239	0.00000	0.00001	0.48559	0.36483	0.64633	**
		T>G	-0.70736	0.00001	0.00004	0.49295	0.36056	0.67395	**
	D9 (Greater Poland Cancer Centre)	C>A	-0.91726	0.00466	0.00559	0.39961	0.21365	0.74746	**
		C>G	-0.94075	0.00221	0.00479	0.39033	0.21541	0.70730	**
		C>T	-1.36801	0.00239	0.00479	0.25461	0.10742	0.60351	**
		T>A	-1.02885	0.00398	0.00559	0.35742	0.17855	0.71545	**
		T>C	-1.15950	0.00013	0.00080	0.31364	0.17463	0.56331	**
		T>G	-0.83633	0.01224	0.01224	0.43330	0.22594	0.83096	*
	EB (Asterand)	C>A	-0.07466	0.79490	0.96969	0.92806	0.52647	1.63598	
		C>G	-0.02680	0.92484	0.96969	0.97356	0.55555	1.70610	
		C>T	-0.41553	0.25558	0.96969	0.65999	0.32168	1.35409	
		T>A	-0.14727	0.66892	0.96969	0.86306	0.43702	1.70441	
		T>C	-0.20839	0.47667	0.96969	0.81189	0.45589	1.44589	
		T>G	-0.01155	0.96969	0.96969	0.98852	0.54323	1.79881	
	ER	C>A	-0.52970	0.01328	0.01593	0.58878	0.38883	0.89155	*

	(University of Pittsburgh)	C>G	-0.15628	0.41523	0.41523	0.85532	0.58516	1.25021	
		C>T	-0.82848	0.00058	0.00116	0.43671	0.27386	0.69640	**
		T>A	-0.75814	0.00047	0.00116	0.46854	0.30805	0.71264	**
		T>C	-0.68040	0.00025	0.00116	0.50641	0.35349	0.72549	**
		T>G	-0.53354	0.00894	0.01340	0.58653	0.39398	0.87317	*
	FR (University of North Carolina)	C>A	-0.21953	0.55315	0.55315	0.80289	0.38722	1.66480	
		C>G	-0.58731	0.11834	0.35502	0.55582	0.26566	1.16288	
		C>T	-0.46305	0.33986	0.40783	0.62936	0.24233	1.63453	
		T>A	-0.76503	0.08575	0.35502	0.46532	0.19423	1.11478	
		T>C	-0.40280	0.27869	0.40783	0.66845	0.32184	1.38833	
		T>G	-0.45463	0.26299	0.40783	0.63469	0.28569	1.41003	
	FS (Essen)	C>A	-0.70637	0.00005	0.00015	0.49343	0.35234	0.69102	**
		C>G	-0.64880	0.00027	0.00041	0.52267	0.37017	0.73800	**
		C>T	-0.85222	0.00021	0.00041	0.42647	0.27299	0.66622	**
		T>A	-0.73975	0.00038	0.00046	0.47723	0.31880	0.71440	**
		T>C	-0.72908	0.00004	0.00015	0.48235	0.34216	0.67998	**
		T>G	-0.61116	0.00136	0.00136	0.54272	0.37440	0.78671	**
	FW (International Genomics Consortium)	C>A	0.25854	0.59444	0.85858	1.29504	0.48931	3.42753	
		C>G	0.10614	0.85160	0.85858	1.11198	0.34630	3.57062	
		C>T	0.66240	0.29433	0.85858	1.93945	0.55112	6.82519	
		T>A	0.11288	0.82805	0.85858	1.11950	0.39828	3.14673	
		T>C	-0.36218	0.40079	0.85858	0.69616	0.29708	1.63135	
		T>G	-0.07916	0.85858	0.85858	0.92390	0.38447	2.22018	
	GN (Roswell)	C>A	0.18300	0.50589	0.87729	1.20081	0.69864	2.06394	
		C>G	0.30774	0.26020	0.87729	1.36035	0.79444	2.32939	
		C>T	0.14352	0.69889	0.87729	1.15433	0.55585	2.39719	
		T>A	0.17938	0.57288	0.87729	1.19647	0.63969	2.23788	
		T>C	0.07716	0.78208	0.87729	1.08022	0.62362	1.87112	
		T>G	0.04638	0.87729	0.87729	1.04747	0.57977	1.89246	

Supplementary Table 3. Output from negative binomial regressions with the Yale Melanoma project cohort, with separate models fitted for each of the six single nucleotide variant (SNV) classes (outcome is SNV count). In these models, the exponential of the estimated coefficient, the incident rate ratio, can be interpreted as a multiplicative factor affecting the expected SNV count outcome variable. The accompanying confidence intervals are at 95%, and stars indicate significance at thresholds: 0.10 (^), 0.05 (*) and 0.01 (**).

Variable	Categorical variable level	Outcome	Coefficient estimate	P-value	Corrected p-value (BH)	exp(Coefficient)	Confidence interval for exp(Coefficient)		
Intercept		C>A	0.82594	0.05306	0.10612	2.28403	0.98914	5.27408	
		C>G	0.66420	0.11443	0.17165	1.94293	0.85174	4.43208	
		C>T	3.91200	0.00000	0.00000	49.99895	18.80097	132.96630	
		T>A	0.24460	0.59890	0.59890	1.27711	0.51332	3.17735	
		T>C	1.38480	0.00345	0.01034	3.99401	1.57903	10.10245	
		T>G	0.29836	0.56285	0.59890	1.34765	0.49050	3.70265	
R allele presence		C>A	0.39330	0.00766	0.02120	1.48186	1.10986	1.97854	*
		C>G	0.29941	0.03787	0.04544	1.34906	1.01691	1.78971	*
		C>T	0.45556	0.01060	0.02120	1.57706	1.11209	2.23643	*
		T>A	0.38009	0.01770	0.02655	1.46242	1.06830	2.00192	*
		T>C	0.45189	0.00716	0.02120	1.57128	1.13035	2.18422	*
		T>G	0.33657	0.06226	0.06226	1.40013	0.98289	1.99449	^
Age at diagnosis (years)		C>A	0.02312	0.00005	0.00010	1.02338	1.01204	1.03486	**
		C>G	0.01744	0.00182	0.00182	1.01760	1.00650	1.02882	**
		C>T	0.02967	0.00001	0.00003	1.03012	1.01657	1.04384	**
		T>A	0.03224	0.00000	0.00000	1.03277	1.02031	1.04537	**
		T>C	0.02381	0.00019	0.00029	1.02410	1.01136	1.03700	**
		T>G	0.02451	0.00040	0.00048	1.02481	1.01100	1.03881	**
Gender (baseline level: female)	Male	C>A	0.08888	0.57832	0.82146	1.09295	0.79890	1.49522	
		C>G	-0.04533	0.77090	0.82146	0.95568	0.70437	1.29665	
		C>T	0.16542	0.38630	0.80177	1.17989	0.81153	1.71544	
		T>A	0.16430	0.34440	0.80177	1.17856	0.83837	1.65681	
		T>C	-0.04059	0.82146	0.82146	0.96022	0.67495	1.36607	
		T>G	0.16304	0.40089	0.80177	1.17708	0.80464	1.72191	
Tissue type (baseline level: primary tumour)	Metastasis	C>A	-0.13259	0.46647	0.85740	0.87582	0.61296	1.25141	
		C>G	-0.04082	0.81754	0.86435	0.96000	0.67868	1.35793	
		C>T	-0.12458	0.57160	0.85740	0.88287	0.57340	1.35936	
		T>A	-0.16870	0.39330	0.85740	0.84476	0.57349	1.24435	
		T>C	-0.15419	0.45707	0.85740	0.85711	0.57089	1.28683	
		T>G	-0.03796	0.86435	0.86435	0.96275	0.62281	1.48823	

Supplementary Table 4. Output from negative binomial regressions with the combined TCGA data and Yale Melanoma project cohort data, with separate models fitted for each of the six single nucleotide variant (SNV) classes (outcome is SNV count) adjusted for the clinical covariates in common between the two datasets. In these models, the exponential of the estimated coefficient, the incident rate ratio, can be interpreted as a multiplicative factor affecting the expected SNV count outcome variable. The accompanying confidence intervals are at 95%, and stars indicate significance at thresholds: 0.10 (ˆ), 0.05 (*) and 0.01 (**).

Variable	Categorical variable level	Outcome	Coefficient estimate	P-value	Corrected p-value (BH)	exp(Coefficient)	Confidence interval for exp(Coefficient)		
Intercept		C>A	1.76000	0.00000	0.00000	5.81242	3.40597	9.91912	
		C>G	1.93111	0.00000	0.00000	6.89715	3.97655	11.96280	
		C>T	5.41069	0.00000	0.00000	223.78537	115.03298	435.35246	
		T>A	1.91108	0.00000	0.00000	6.76042	3.70004	12.35210	
		T>C	2.11325	0.00000	0.00000	8.27506	4.63282	14.78079	
		T>G	1.09271	0.00037	0.00037	2.98236	1.64069	5.42118	
R allele presence		C>A	0.33914	0.00010	0.00020	1.40374	1.18499	1.66288	**
		C>G	0.24172	0.00687	0.00687	1.27343	1.06915	1.51675	**
		C>T	0.35227	0.00129	0.00194	1.42230	1.14870	1.76106	**
		T>A	0.28007	0.00461	0.00553	1.32322	1.09072	1.60527	**
		T>C	0.46729	0.00000	0.00001	1.59566	1.32590	1.92030	**
		T>G	0.39894	0.00005	0.00016	1.49024	1.22984	1.80577	**
Age at diagnosis (years)		C>A	0.01480	0.00000	0.00000	1.01491	1.00920	1.02066	**
		C>G	0.00826	0.00534	0.00534	1.00829	1.00247	1.01415	**
		C>T	0.01648	0.00001	0.00001	1.01662	1.00943	1.02386	**
		T>A	0.01578	0.00000	0.00000	1.01590	1.00940	1.02244	**
		T>C	0.01779	0.00000	0.00000	1.01795	1.01169	1.02424	**
		T>G	0.02022	0.00000	0.00000	1.02043	1.01392	1.02698	**
Gender (baseline level: female)	Male	C>A	0.09798	0.27515	0.33018	1.10294	0.92469	1.31556	
		C>G	-0.14659	0.11135	0.22270	0.86365	0.72095	1.03460	
		C>T	0.21119	0.06159	0.18476	1.23515	0.98975	1.54139	
		T>A	0.07321	0.47271	0.47271	1.07596	0.88069	1.31453	
		T>C	0.14083	0.15016	0.22525	1.15123	0.95008	1.39498	
		T>G	0.23438	0.02162	0.12972	1.26413	1.03518	1.54372	
Tissue type (baseline level: primary tumour)	Metastasis	C>A	0.19705	0.18096	0.30604	1.21781	0.91200	1.62616	
		C>G	0.16007	0.29645	0.35574	1.17359	0.86834	1.58615	
		C>T	0.08014	0.65854	0.65854	1.08344	0.75864	1.54728	
		T>A	0.25038	0.12885	0.30604	1.28451	0.92949	1.77513	
		T>C	0.27562	0.08176	0.30604	1.31735	0.96568	1.79708	
		T>G	0.20757	0.20403	0.30604	1.23068	0.89296	1.69612	
Tissue	D3 (MD	C>A	-0.43590	0.00302	0.00302	0.64668	0.48528	0.86176	**

collection centre (baseline level: University of Sydney)	Anderson)	C>G	-0.44854	0.00300	0.00302	0.63856	0.47528	0.85793	**
		C>T	-0.63982	0.00059	0.00089	0.52739	0.36682	0.75825	**
		T>A	-0.65240	0.00011	0.00033	0.52079	0.37501	0.72324	**
		T>C	-0.65263	0.00006	0.00033	0.52067	0.37968	0.71403	**
		T>G	-0.60988	0.00030	0.00060	0.54342	0.39117	0.75493	**
	D9 (Greater Poland Cancer Centre)	C>A	-0.68239	0.01285	0.01927	0.50541	0.29549	0.86445	*
		C>G	-0.38389	0.17441	0.17441	0.68120	0.39116	1.18630	
		C>T	-1.00726	0.00330	0.01341	0.36522	0.18691	0.71362	*
		T>A	-0.85674	0.00670	0.01341	0.42454	0.22885	0.78758	*
		T>C	-0.82432	0.00585	0.01341	0.43853	0.24435	0.78701	*
		T>G	-0.57179	0.06527	0.07832	0.56451	0.30731	1.03699	^
	EB (Asterand)	C>A	-0.12927	0.58168	0.58168	0.87873	0.55390	1.39406	
		C>G	-0.18354	0.45664	0.58168	0.83232	0.51239	1.35200	
		C>T	-0.49404	0.09613	0.54023	0.61016	0.34077	1.09252	
		T>A	-0.22619	0.39203	0.58168	0.79757	0.47453	1.34053	
		T>C	-0.34333	0.18008	0.54023	0.70940	0.42903	1.17300	
		T>G	-0.17093	0.50929	0.58168	0.84288	0.50666	1.40219	
	ER (University of Pittsburgh)	C>A	-0.44604	0.01365	0.02002	0.64016	0.44934	0.91201	*
		C>G	-0.22277	0.22468	0.22468	0.80030	0.55826	1.14727	
		C>T	-0.76483	0.00091	0.00273	0.46541	0.29686	0.72968	**
		T>A	-0.70945	0.00063	0.00273	0.49191	0.32816	0.73738	**
		T>C	-0.62625	0.00162	0.00324	0.53459	0.36274	0.78788	**
		T>G	-0.48940	0.01668	0.02002	0.61300	0.41080	0.91471	*
	FR (University of North Carolina)	C>A	-0.33540	0.35863	0.35863	0.71505	0.34893	1.46535	
		C>G	-0.74510	0.05384	0.16151	0.47469	0.22256	1.01244	
		C>T	-0.53614	0.23821	0.28586	0.58500	0.23967	1.42789	
		T>A	-0.84420	0.04642	0.16151	0.42990	0.18732	0.98663	
		T>C	-0.47763	0.22586	0.28586	0.62025	0.28599	1.34518	
		T>G	-0.51840	0.21474	0.28586	0.59547	0.26222	1.35226	
	FS (Essen)	C>A	-0.65956	0.00012	0.00072	0.51708	0.37038	0.72187	**
		C>G	-0.63668	0.00031	0.00094	0.52904	0.37502	0.74634	**
		C>T	-0.68544	0.00132	0.00199	0.50387	0.33217	0.76431	**
		T>A	-0.60030	0.00187	0.00225	0.54864	0.37635	0.79983	**
		T>C	-0.61629	0.00092	0.00184	0.53994	0.37568	0.77603	**
		T>G	-0.48619	0.01148	0.01148	0.61497	0.42209	0.89599	*
	FW (International Genomics Consortium)	C>A	0.84999	0.02444	0.07333	2.33962	1.11648	4.90273	^
		C>G	0.64258	0.09497	0.18994	1.90138	0.89385	4.04457	
		C>T	1.73909	0.00051	0.00307	5.69217	2.14707	15.09068	**
		T>A	0.63649	0.14314	0.21471	1.88983	0.80538	4.43451	

		T>C	0.21540	0.60777	0.60777	1.24035	0.54387	2.82874	
		T>G	0.40862	0.34135	0.40962	1.50474	0.64747	3.49704	
	GN (Roswell)	C>A	0.01322	0.96072	0.97542	1.01331	0.59799	1.71706	
		C>G	-0.00844	0.97542	0.97542	0.99159	0.57862	1.69931	
		C>T	0.06990	0.83953	0.97542	1.07240	0.54422	2.11319	
		T>A	0.06316	0.83662	0.97542	1.06520	0.58350	1.94457	
		T>C	-0.11681	0.69385	0.97542	0.88975	0.49667	1.59393	
		T>G	-0.13579	0.65685	0.97542	0.87303	0.47889	1.59155	
	Yale	C>A	-0.51762	0.00003	0.00003	0.59594	0.46825	0.75845	**
		C>G	-0.54331	0.00002	0.00003	0.58083	0.45283	0.74501	**
		C>T	-0.65565	0.00003	0.00003	0.51910	0.38220	0.70504	**
		T>A	-0.59425	0.00003	0.00003	0.55198	0.41963	0.72607	**
		T>C	-0.57225	0.00003	0.00003	0.56425	0.43334	0.73472	**
		T>G	-0.65883	0.00000	0.00002	0.51746	0.39398	0.67962	**

Supplementary Table 5. Credibility intervals (95%) for the prevalence of each signature in the zero *R* allele group and in the one or two *R* allele group. The significant difference in exposure to signature 3 is highlighted.

		Extracted signature								
		0	1	2	3	4	5	6	7	8
Zero R allele group	Lower bound	0.010	0.580	0.000	0.061	0.000	0.007	0.003	0.000	0.000
	Upper bound	0.067	0.889	0.257	0.093	0.014	0.020	0.014	0.007	0.009
One or two R allele group	Lower bound	0.007	0.586	0.000	0.036	0.000	0.010	0.002	0.000	0.002
	Upper bound	0.064	0.919	0.283	0.059	0.009	0.022	0.014	0.004	0.008

Supplementary Table 6. Output from negative binomial regressions with the TCGA data classifying Thr95Met and Arg213Trp as *r* and *R* alleles respectively, with separate models fitted for each of the six single nucleotide variant (SNV) classes (outcome is SNV count). In these models, the exponential of the estimated coefficient, the incident rate ratio, can be interpreted as a multiplicative factor affecting the expected SNV count outcome variable. The accompanying confidence intervals are at 95%, and stars indicate significance at thresholds: 0.10 (^), 0.05 (*) and 0.01 (**).

Variable	Categorical variable level	Outcome	Coefficient estimate	P-value	Corrected p-value (BH)	exp(Coefficient)	Confidence interval for exp(Coefficient)		
Intercept		C>A	1.409487696	0.001133883	0.001700824	4.093857566	1.775834355	9.437631233	
		C>G	0.660220822	0.112612197	0.112612197	1.935219625	0.854322139	4.383680145	
		C>T	5.207789362	0	0	182.6897506	62.77367026	531.680637	
		T>A	1.594328239	0.00075966	0.001519319	4.925019522	1.965472106	12.34096237	
		T>C	2.274857377	5.52E-06	1.66E-05	9.726531682	3.8910005	24.31390553	
		T>G	0.92571307	0.043109981	0.051731977	2.523667171	1.029576309	6.185938757	
R allele presence		C>A	0.252829239	0.014234107	0.021351161	1.287663375	1.052506045	1.575360991	*
		C>G	0.299644383	0.003782157	0.021351161	1.34937886	1.102924941	1.650904102	*
		C>T	0.240890169	0.078518251	0.078518251	1.272381281	0.972667986	1.664446807	^
		T>A	0.221518575	0.060580838	0.072697006	1.247970429	0.990124062	1.572964694	^
		T>C	0.282799362	0.007249543	0.021351161	1.326838921	1.080212991	1.629772588	*
		T>G	0.281690874	0.013175986	0.021351161	1.325368951	1.06138762	1.655005978	*
Age at diagnosis (years)		C>A	0.008974606	0.007388709	0.012894394	1.009014998	1.00243616	1.015637012	*
		C>G	0.009794468	0.003559414	0.010678241	1.009842591	1.003252036	1.016476441	*
		C>T	0.006922696	0.112691722	0.112691722	1.006946713	0.998357627	1.015609693	
		T>A	0.009167763	0.022015407	0.026418488	1.009209916	1.001337292	1.017144436	*
		T>C	0.009044268	0.008596263	0.012894394	1.009085291	1.0023253	1.015890873	*
		T>G	0.012700397	0.000546157	0.003276945	1.01278139	1.005578987	1.02003538	**
Breslow thickness (mm)		C>A	0.026223853	0.002438076	0.007314228	1.026570724	1.009522519	1.043906829	**
		C>G	0.040212768	6.54E-06	3.93E-05	1.041032249	1.023466215	1.058899773	**
		C>T	0.024227978	0.081060192	0.107639964	1.02452386	0.996867444	1.052947557	
		T>A	0.017976276	0.073845982	0.107639964	1.018138822	0.998243538	1.038430623	
		T>C	0.017139369	0.100145287	0.107639964	1.017287091	0.99654443	1.038461501	
		T>G	0.015325362	0.107639964	0.107639964	1.015443397	0.9966155	1.034626989	
Gender (baseline level: female)	Male	C>A	0.188962829	0.078316107	0.208408656	1.207996049	0.978655912	1.491080202	
		C>G	-0.001645654	0.988065427	0.988065427	0.998355699	0.803653229	1.240229076	
		C>T	0.233983629	0.104204328	0.208408656	1.263623806	0.952460405	1.676442521	
		T>A	0.139202466	0.270055798	0.389521376	1.149356782	0.896777018	1.473076344	
		T>C	0.110317036	0.324601147	0.389521376	1.116632027	0.895823878	1.39186632	
		T>G	0.209958219	0.070174095	0.208408656	1.233626516	0.982720768	1.548592877	

Clark level (baseline level: V)	Level I	C>A	0.760364082	0.040891112	0.158200535	2.139054871	1.032694572	4.430696031	
		C>G	0.719080613	0.052733512	0.158200535	2.05254526	0.991325538	4.249806832	
		C>T	0.619619832	0.222019911	0.222019911	1.858221472	0.682452814	5.05967148	
		T>A	0.61918744	0.154752366	0.222019911	1.857418165	0.788906459	4.373144876	
		T>C	0.522054239	0.220583601	0.222019911	1.685486488	0.723733733	3.925289882	
		T>G	0.638824295	0.120203082	0.222019911	1.894252488	0.843867829	4.252078779	
	Level II	C>A	0.297287047	0.33808009	0.374027717	1.346201667	0.725116076	2.49926734	
		C>G	0.273359655	0.277717164	0.374027717	1.31437288	0.800971785	2.156850093	
		C>T	0.434422734	0.261112546	0.374027717	1.544071462	0.717043132	3.324983637	
		T>A	0.289877438	0.374027717	0.374027717	1.336263703	0.700764401	2.548075617	
		T>C	0.346899397	0.21983579	0.374027717	1.414674397	0.809584756	2.472012517	
		T>G	0.365552651	0.244472857	0.374027717	1.441310329	0.773937802	2.684163327	
	Level III	C>A	0.597994316	0.01379713	0.041391391	1.818467869	1.135789149	2.911478239	*
		C>G	0.509199764	0.011946481	0.041391391	1.663959102	1.120429573	2.471159241	*
		C>T	0.598371568	0.036630231	0.066446693	1.819154019	1.038761928	3.185832342	^
		T>A	0.56155263	0.044297795	0.066446693	1.753392757	1.014982562	3.029003922	^
		T>C	0.279930931	0.328519105	0.328519105	1.323038428	0.742045145	2.358927477	
		T>G	0.427612468	0.089684198	0.107621038	1.533591649	0.93392841	2.518290825	
	Level IV	C>A	0.570253192	0.003698389	0.011095168	1.768714819	1.211336418	2.582562583	*
		C>G	0.513838417	0.00231944	0.011095168	1.67169556	1.203872998	2.321313003	*
		C>T	0.555395021	0.032056506	0.048084759	1.742629223	1.050504758	2.8907595	*
		T>A	0.558184134	0.021710314	0.043420629	1.747496399	1.089065049	2.804004835	*
		T>C	0.219205454	0.331527599	0.331527599	1.245087058	0.791525672	1.958548963	
		T>G	0.386880921	0.095459729	0.114551675	1.472381151	0.931260658	2.327926382	
Ulceration (baseline level: no)	Yes	C>A	-0.151799693	0.339607991	0.961058596	0.859160359	0.624403726	1.18217828	
		C>G	-0.094617277	0.510675288	0.961058596	0.909721039	0.682402064	1.212763579	
		C>T	0.000853822	0.995935616	0.995935616	1.000854186	0.718473523	1.394218535	
		T>A	-0.070555162	0.640705731	0.961058596	0.931876334	0.69068647	1.25729045	
		T>C	-0.120003777	0.332816565	0.961058596	0.886917087	0.694950672	1.131910437	
		T>G	-0.01571313	0.921022719	0.995935616	0.984409677	0.716603708	1.352298909	
Body area (baseline level: extremities)	Head and neck	C>A	0.484147716	0.012120662	0.012120662	1.622791341	1.113227662	2.365600341	*
		C>G	0.69235248	0.000195296	0.000585888	1.998411231	1.394410064	2.864040895	**
		C>T	0.855911163	0.002260072	0.002712086	2.353517843	1.367795355	4.049616208	**
		T>A	0.77659869	0.001890708	0.002712086	2.174065006	1.342195116	3.521513818	**
		T>C	0.921531894	4.75E-05	0.000284921	2.513137304	1.639637442	3.851985168	**
		T>G	0.671194354	0.001188159	0.002376317	1.956572767	1.308035499	2.926661391	**
	Trunk	C>A	-0.291395433	0.011842656	0.071055937	0.747220144	0.596039394	0.936746715	^

		C>G	-0.10628563	0.341035308	0.341035308	0.899167782	0.721839713	1.120058491	
		C>T	-0.250369218	0.098431758	0.176016713	0.778513289	0.578186918	1.048247413	
		T>A	-0.225381979	0.115898791	0.176016713	0.79821126	0.602054266	1.058278716	
		T>C	-0.183788958	0.123526928	0.176016713	0.832111396	0.658171233	1.052020113	
		T>G	-0.188349956	0.146680595	0.176016713	0.828324779	0.641699536	1.069226174	
Tissue type (baseline level: primary tumour)	Distant metastasis	C>A	0.729479943	0.009089361	0.018178722	2.074001729	1.201928367	3.578818248	*
		C>G	0.766379857	0.006793693	0.018178722	2.151961727	1.24011275	3.734288898	*
		C>T	0.652079861	0.079770834	0.079770834	1.919529033	0.924534779	3.985346785	^
		T>A	0.792023732	0.016674692	0.020009631	2.207860025	1.157042686	4.213021655	*
		T>C	0.808335716	0.003026395	0.018158373	2.244169942	1.319364255	3.81721629	*
		T>G	0.720979315	0.015312329	0.020009631	2.056446132	1.150081723	3.677104516	*
	Regional (sub) cutaneous tissue	C>A	0.546251724	0.044993302	0.061097938	1.726768468	1.012443656	2.945081759	^
		C>G	0.632033451	0.021812802	0.061097938	1.881432494	1.098064393	3.22366179	^
		C>T	0.546251222	0.123000675	0.123000675	1.7267676	0.860910198	3.463458036	
		T>A	0.613937441	0.050914948	0.061097938	1.847692274	0.997527769	3.422427773	^
		T>C	0.630785638	0.017260535	0.061097938	1.879086282	1.119426586	3.154262456	^
		T>G	0.61127043	0.034417406	0.061097938	1.842771024	1.046382232	3.24528164	^
	Regional lymph node	C>A	0.29786888	0.236025961	0.283231153	1.34698516	0.821363102	2.208973129	
		C>G	0.590558381	0.029595521	0.177573125	1.804996009	1.061818481	3.068331028	
		C>T	0.27423178	0.414675331	0.414675331	1.315519678	0.677551582	2.554184908	
		T>A	0.384286581	0.213023431	0.283231153	1.468566244	0.799033243	2.699120259	
		T>C	0.350448936	0.156432782	0.283231153	1.419704763	0.873318069	2.307935314	
		T>G	0.475592368	0.078846703	0.236540109	1.608967016	0.946104928	2.736244979	
Tissue collection centre (baseline level: University of Sydney)	D3 (MD Anderson)	C>A	-0.430119547	0.003520278	0.004224334	0.650431333	0.488061969	0.866818039	**
		C>G	-0.37380352	0.014198729	0.014198729	0.688112099	0.511008138	0.92659632	*
		C>T	-0.762504507	5.42E-05	8.12E-05	0.466496619	0.323676337	0.67233551	**
		T>A	-0.704683261	5.25E-05	8.12E-05	0.494265103	0.353164015	0.691740894	**
		T>C	-0.730349424	1.46E-06	8.76E-06	0.481740629	0.36025007	0.644202605	**
		T>G	-0.698676038	1.37E-05	4.10E-05	0.497243199	0.364844583	0.677688009	**
	D9 (Greater Poland Cancer Centre)	C>A	-0.945200506	0.003141097	0.004711646	0.388601646	0.209329697	0.721403803	**
		C>G	-1.059556058	0.001968831	0.004065921	0.346609651	0.179786638	0.668226801	**
		C>T	-1.352480218	0.005214075	0.00625689	0.258598085	0.102412792	0.652974774	**
		T>A	-1.114570108	0.00203296	0.004065921	0.328056277	0.163070183	0.659966885	**
		T>C	-1.075171324	0.00095226	0.004065921	0.341239288	0.182625732	0.637611415	**

		T>G	-0.777190913	0.017421472	0.017421472	0.459695524	0.242737736	0.87056911	*
	EB (Asterand)	C>A	-0.032751967	0.90765746	0.943704658	0.967778571	0.554682811	1.68852422	
		C>G	0.026068369	0.930304329	0.943704658	1.026411121	0.568774844	1.852261576	
		C>T	-0.451262273	0.223250636	0.943704658	0.636823798	0.307313181	1.319645805	
		T>A	-0.207988594	0.54340856	0.943704658	0.8122163	0.413228152	1.596443311	
		T>C	-0.228614332	0.407922938	0.943704658	0.795635326	0.462098479	1.369914856	
		T>G	0.021759624	0.943704658	0.943704658	1.021998091	0.556676204	1.876279409	
	ER (University of Pittsburgh)	C>A	-0.527766394	0.003557415	0.005336123	0.58992115	0.414599778	0.839380488	**
		C>G	-0.167719717	0.345026432	0.345026432	0.845590806	0.596047175	1.199609429	
		C>T	-0.834173318	0.000542491	0.001084982	0.434233306	0.272158011	0.692827536	**
		T>A	-0.74698021	0.000412319	0.001084982	0.473795157	0.314489254	0.71379816	**
		T>C	-0.739738196	4.73E-05	0.00028393	0.477238842	0.33587849	0.678093178	**
		T>G	-0.549005005	0.005154342	0.006185211	0.577524158	0.393882569	0.846785766	**
	FR (University of North Carolina)	C>A	-0.188669875	0.612695328	0.612695328	0.828059825	0.397536494	1.724830513	
		C>G	-0.493107229	0.188754818	0.408122229	0.610725778	0.292243717	1.276283987	
		C>T	-0.453798389	0.340608931	0.408730718	0.635210786	0.249034122	1.620230753	
		T>A	-0.814235367	0.071275794	0.408122229	0.442977913	0.182734431	1.073850344	
		T>C	-0.416212102	0.263063831	0.408122229	0.659540366	0.317520133	1.369971381	
		T>G	-0.438936416	0.272081486	0.408122229	0.644721772	0.293934651	1.41414482	
	FS (Essen)	C>A	-0.696032791	0.000116694	0.000273617	0.498559274	0.351658297	0.706826349	**
		C>G	-0.637046714	0.000322125	0.00038655	0.528851971	0.37517798	0.745471275	**
		C>T	-0.873165735	0.000156809	0.000273617	0.417627357	0.266988264	0.653259461	**
		T>A	-0.761072465	0.000182411	0.000273617	0.46716514	0.315018599	0.692794866	**
		T>C	-0.733046583	4.71E-05	0.000273617	0.480443049	0.339306384	0.680286414	**
		T>G	-0.631803524	0.00079901	0.00079901	0.531632125	0.368551129	0.766875188	**
	FW (International Genomics Consortium)	C>A	0.351263991	0.507077558	0.827031672	1.420862371	0.487762454	4.139002219	
		C>G	0.22797411	0.58031008	0.827031672	1.256052806	0.554662955	2.844373576	
		C>T	0.603800342	0.425864237	0.827031672	1.82905665	0.392582425	8.521645438	
		T>A	0.104236509	0.827031672	0.827031672	1.109862916	0.432231503	2.849851723	
		T>C	-0.357090331	0.391846455	0.827031672	0.69970929	0.307508403	1.592129141	

		T>G	-0.15786199	0.728429507	0.827031672	0.85396763	0.347528853	2.098417746
	GN (Roswell)	C>A	0.200891001	0.530747586	0.891887364	1.222491514	0.646394336	2.312033722
		C>G	0.373770759	0.197823959	0.891887364	1.453203979	0.820332823	2.574323184
		C>T	0.171402909	0.63985129	0.891887364	1.186968893	0.576970037	2.441886168
		T>A	0.214365205	0.515772362	0.891887364	1.239075088	0.646887212	2.373376758
		T>C	0.081502436	0.772238424	0.891887364	1.084915861	0.6229055	1.889600308
		T>G	0.04013989	0.891887364	0.891887364	1.040956384	0.582087667	1.86155841

Supplementary References

1. Davies, J. R. *et al.* Inherited variants in the MC1R gene and survival from cutaneous melanoma: a BioGenoMEL study. *Pigment Cell Melanoma Res.* **25,** 384–394 (2012).
2. Beaumont, K. A. *et al.* Receptor function, dominant negative activity and phenotype correlations for MC1R variant alleles. *Hum. Mol. Genet.* **16,** 2249–2260 (2007).
3. Duffy, D. L. *et al.* Interactive effects of MC1R and OCA2 on melanoma risk phenotypes. *Hum. Mol. Genet.* **13,** 447–461 (2004).
4. Ng, P. C. & Henikoff, S. SIFT: Predicting amino acid changes that affect protein function. *Nucleic Acids Res.* **31,** 3812–3814 (2003).
5. Adzhubei, I., Jordan, D. M. & Sunyaev, S. R. Predicting functional effect of human missense mutations using PolyPhen-2. *Curr. Protoc. Hum. Genet. Editor. Board Jonathan Haines Al* **Chapter 7,** Unit7.20 (2013).
6. Yates, A. *et al.* Ensembl 2016. *Nucleic Acids Res.* **44,** D710–716 (2016).
7. Cancer Genome Atlas Network. Genomic Classification of Cutaneous Melanoma. *Cell* **161,** 1681–1696 (2015).
8. Krauthammer, M. & others. Exome sequencing identifies recurrent mutations in NF1 and RASopathy genes in sun-exposed melanomas. *Nat Genet* **47,** 996-1002 (2015).
9. Forbes, S. A. *et al.* COSMIC: exploring the world’s knowledge of somatic mutations in human cancer. *Nucleic Acids Res.* **43,** D805–811 (2015).
10. Alexandrov, L. B. *et al.* Signatures of mutational processes in human cancer. *Nature* **500,** 415–421 (2013).
